# Distinct patterns of variation in the distribution of knee pain

**DOI:** 10.1038/s41598-018-34950-2

**Published:** 2018-11-08

**Authors:** Shellie A. Boudreau, Albert Cid Royo, Mark Matthews, Thomas Graven-Nielsen, Ernest N. Kamavuako, Greg Slabaugh, Kristian Thorborg, Bill Vicenzino, Michael Skovdal Rathleff

**Affiliations:** 1 Center for Neuroplasticity and Pain (CNAP), Department of Health Science and Technology, Aalborg University, Aalborg, Denmark; 2 The University of Queensland, School of Health and Rehabilitation Sciences, Sports Injuries Rehabilitation and Prevention for Health research unit, CCRE Spine, Brisbane, Australia; 3 Sports and Exercise Sciences Research Institute, School of Sport, Ulster University, Belfast, United Kingdom; 4 Center for Robotics Research (CORE), Department of Informatics, King’s College London, London, United Kingdom; 5 Department of Computer Science, City, University of London, London, United Kingdom; 6 Sports Orthopedic Research Center - Copenhagen (SORC-C), Department of Orthopaedic Surgery, Copenhagen University Hospital, Amager-Hvidovre, Denmark; 7 Research Unit for General Practice in Aalborg, Department of Clinical Medicine, Aalborg University, Aalborg, Denmark; 8 SMI, Department of Health Science and Technology, Aalborg University, Aalborg, Denmark

## Abstract

The patient’s expression of pain using digital-body maps expands analytic opportunities for exploring the spatial variation of bodily pain. A common knee pain condition in adolescents and adults is patellofemoral pain (PFP) and recently PFP was shown to be characterized by a heterogeneous distribution of pain. Whether there are important patterns in these distributions remains unclear. This pioneering study assesses the spatial variation of pain using principal component analysis and a clustering approach. Detailed digital-body maps of knee pain were drawn by 299 PFP patients of mixed sex, age, and pain severity. Three pain distribution patterns emerged resembling an Anchor, Hook, and an Ovate shape on and around the patella. The variations in pain distribution were independent of sex, age, and pain intensity. Bilateral pain associated with a longer duration of pain and the majority characterized by the Hook and Ovate pain distributions. Bilateral and/or symmetrical pain between the left and right knees may represent symptoms associated with longstanding PFP. The distinct patterns of pain location and area suggest specific underlying structures cannot be ruled out as important drivers, although central neuronal mechanisms possibly exemplified by the symmetrical representation of pain may play a role in individuals with longstanding symptoms.

## Introduction

The possibility for patients to digitally express where they experience pain expands the analytic opportunities for understanding the spatial variation and distribution of their pain experience. The location and area of pain drawn relative to visible anatomical features on a body chart reflect pain distribution. Assessing pain distribution by using pain drawings is a clinically relevant and reliable method^1–8^. Pain drawings are known as discomfort drawings, pain charts, and pain maps and used to achieve a better understanding of a disease or condition^7,9–13^. Pain maps can potentially provide insight into the underlying pathology where pain severity so far has contributed inadequately.

The distribution of patellofemoral pain (PFP), one of the most common knee pain presentations, is traditionally described as pain centrally (central patellar) or around (peripatellar) the patella. Patellofemoral pain is a condition considered to have a common genesis in adolescence and affects one in fourteen adolescents and up to one in five adults^14^. Moreover, PFP is a known recurrent pain condition and up to 50% will continue to experience pain even years later^15,16^. The diversity of pain distribution in patients suffering from PFP^17^ suggests that central patellar and peripatellar may be too crude descriptions of PFP distribution^17^. Especially considering that several structures around the knee, such as the medial and lateral retinaculum, the patellar subchondral bone, the synovium, the infra-patellar fat pad are all capable of producing knee pain^18^ as well as pain mechanisms with-in the central nervous system^19^ likely contribute to the diversity in PFP distribution.

Digital pain mapping has led to several insights describing the clinical picture of PFP. The first is the identification of highly symmetric pain drawings between the left and right knee for individuals with bilateral PFP^17^. In addition, the area of pain tended to be larger for individuals with longer duration of pain symptoms, suggesting that pain may spread with extended symptom duration. A superimposed overlay of all individual pain drawings further suggested that the distribution of pain progressed from inferior to the superior patellar areas similar to a small ‘u’ to an ‘O’ shape on and around the patella^17^. A limitation of this earlier study is that it observed a small number of adult patients. Whether important patterns of variation in the pain distribution exist and how these patterns of variability relate to the structures capable of driving pain is uncertain. Further, the nature of the relationship between pain distribution and duration of pain symptoms is unclear.

The purpose of this study was to explore and identify patterns in the spatial variation of pain distribution as drawn by patients with PFP on a digital body chart of the knee. Applying advanced information processing approaches for analyzing the variations in the distribution of bodily pain as an ‘image’ or pain map has yet to be performed. Thus, as a first step towards understanding PFP distributions, the aim of this study was to identify patterns and examine the variation in pain distribution by using principal component analysis and K-means clustering. Secondary aims consisted of exploring the relationship between patterns of variation in pain distribution and pain duration, pain intensity, laterality (bilateral vs. unilateral) and pain symmetry in those with bilateral pain. Other factors such as age and sex were also investigated as explanatory variables for both aims.

## Methods

### Participants

Patellofemoral pain map analysis is based on a combined cohort of 299 patients with PFP stemming from baseline examinations from two separate intervention studies in patients with PFP. One hundred and sixty nine PFP maps originated from 218 participants (age range 18–40 years) recruited from Australia and Denmark into the Foot Orthoses versus Hip Exercises (FOHX) study^20^, with matching patient reported outcomes available for analyses. The Multidimensional Diagnostics and Effect of Activity Modification in Young Adolescents With Patellofemoral Pain (ACTMOD) study from Denmark recruited 151 adolescents between 10 and 14 years of age with PFP and 131 PFP maps with matching patient reported outcomes were available at the time of this study.

In both cohorts, patients were recruited from social media and advertisements. Volunteers who expressed interest were screened through a verbal screening process and then invited to a clinical examination to determine eligibility. During the clinical examination the inclusion criteria for PFP was insidious onset of anterior knee or central patellar pain of more than six weeks duration and provoked by at least two of the following daily activities: (1) Prolonged sitting or kneeling, squatting, running, hopping, or stair walking; (2) tenderness on palpation of the patella, pain when stepping down or double leg squatting; and (3) worst pain during the previous week of more than 3 cm on a 10 cm visual analogue scale (VAS; 0 identified ‘no pain’ and 10 cm anchored ‘worse pain imaginable’). Exclusion criteria were concomitant injury or pain from the hip, lumbar spine, or other knee structures; previous knee surgery; self-reported patellofemoral instability; knee joint effusion^21^.

The study was conducted in accordance with the Helsinki Declaration and was approved by the local ethics committee in the North Denmark Region (N-20140100) and the University of Queensland Medical Research Ethics Committee (2013000981). Adolescents below 18 years of age were required to give informed consent together with parental consent. Young adults aged 18 or 19 years were allowed to give informed consent without parental consent and adults gave written informed consent.

### Collection of Patient Reported Outcomes

In addition to age and sex, the patient reported outcomes for this study consisted of: (1) worse pain in the knee(s) over the last 24 hours on a 0–10 numeric rating scale (NRS; 0 being ‘no pain’ and 10 ‘worse pain imaginable’), (2) symptom duration (months), and (3) unilateral or bilateral pain (yes/no). Patients in the FOHX trial and ACTMOD study all completed the self-report outcomes at the time of inclusion into the study.

### Creation of Pain Maps and Drawing Instructions

Pain area and location were drawn on high-resolution and detailed digital-body chart using the Navigate Pain Android app (Aalborg University, Version 1) and the digital-body-chart has been validated against paper drawings for patellofemoral pain^8^ among other conditions^2,3^. Participants drew with a Samsung Galaxy Note accessory stylus ‘S pen’ on a Samsung Galaxy Note 10.1 tablet (Android 4.1.2). The drawing is accurate to the tip size of the stylus which is approximately 1.5 mm. Similar to our previous studies^2,3,17^ the instructions to participants consisted of (1) draw one or more areas of their current pain on and around the knee, (2) draw as accurately as possible and to the best of your ability, and (3) shade pain areas completely and avoid using circle outlines or cross-marks for pain areas.

**Figure 1:**
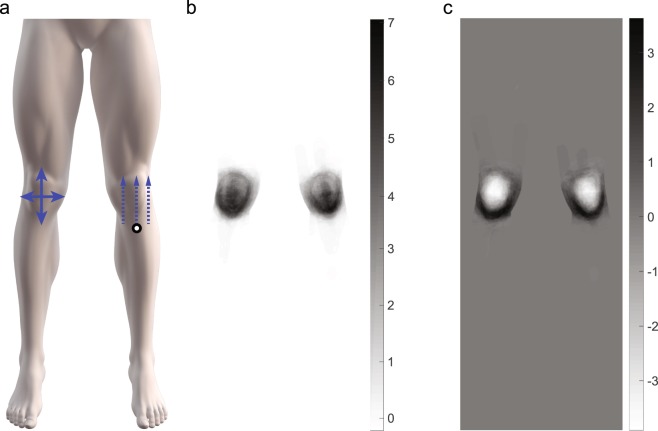
The lower-body template showing the direction of pain area variance (solid arrows) in the proximal and medial to lateral variance directions and a representation of the maximum pain distance (dashed arrow) relative to the tuberositas tibiae (**a**) Eigenvectors of the principle components (PC) showing the two most prevalent PCs (PC1 and PC2). Note the difference in scale and that PC1 is only positive and PC2 is positive and negative indicating spatially anisotropic pain.

Participants drew their pain on a body chart depicting both the left and right knee and thus created a two-knee PFP map. The two-knee map may reflect unilateral or bilateral knee pain. A split body approach in which each bilateral pain drawing contributes with two independent pain maps was evaluated as an option to increase the sample size and reduce the PFP image dimensions. However, the allocation of associated demographics such as age, sex, pain duration, or intensity would repeat with either two distinctly different or completely similar (i.e. symmetric) left and right PFP pain drawings. In consideration of our previous study^17^ bilateral symmetrical knee pain was the norm rather than the exception and supports the view that for PFP the knees should be viewed as a functional unit. On these bases, the raw two-knee image was selected as the best approach for exploring the spatial variation in PFP distribution. Some of the PFP maps from the Australian FOHX cohort were recorded on a full-body view template whilst those collected from Danish FOHX and ACTMOD cohorts were acquired using a lower-body view template. The PFP maps acquired on the full-body view template (N = 35) were cropped to match the PFP maps acquired using the lower-body view template (Fig. 1a). All images were down sampled to the same resolution (1136 × 447) which does not interfere with the analyses, but it does create a possible limitation of a relatively smaller view of the knee during drawing. Notably, the full-body charts used for this study were displayed on average 3–4 times larger in size than drawings on paper in conventional questionnaires^22,23^ providing a considerable amount of space and easily viewable anatomical detail around the knee for support a precise mapping of the pain location.

### Assessment of Spatial Variation

Principal Component Analysis (PCA) is a commonly applied linear transformation of data and used to characterize variation in multidimensional data structures, such as images^24^. In this study, the PCA is applied to pain maps to identify the primary spatial distributions captured in a covariance matrix. The PCA represents the collection of pain maps as a set of eigenvectors (also called eigen-images, after rearranging back from the 1D vector to a rectangular image) of a covariance matrix with same pixel dimension as the pain map. Thus, each mean-removed pain map can be described by superposition of a small set of weighted principal components (PCs). Each weight is an eigenvalue, and each PC is an eigen-image, the latter characterizing how the pain image differs from the mean pain image in a particular direction.

The PCs clearly representing the highest spatial variation stemming from the PCA of all pain maps were selected for K-means clustering. K-means clustering is a method whereby the data (i.e. weights along the PCs) are divided into clusters to minimize the within cluster distance^21^. The Elbow method^25^ was used to determine the number of clusters. This method plots the intra-cluster variance versus the number of clusters. The point at which the plot exhibits a strong bend, known as the elbow, gives the optimal number of clusters that compactly capture the intra-cluster variance with a minimal number of clusters. The number of clusters K was defined after calculating the mean of the within-cluster sums of point-to-centroid distances. PCA and K-means clustering was performed using Matlab® (version R2017b, Natick, MA, USA).

### Pain Maps and Principal Component Overlay Images

The pain map overlay images for the clusters was created after superimposing the original pain drawing (pain map) acquired from each participant within the respective cluster. The reconstructed pain maps are based on the weighted summation of the selected PCs and presented using green-yellow-blue color-map known as Viridis. Using a Gaussian kernel smoothing function with a variance of five and size of 21 × 21 pixels the common pain areas are given an emphasis. Following smoothing, contour plots using the Matlab® contour function display the most common regions (green = 50% and yellow = 75%) and pain-shape morphology within each cluster. An overlay of the original pain maps is represented using a red color-scheme, reflecting the original drawing color, and consists of a linearly increasing pink (frequency of one) to dark brown (highest frequency).

### Pain Map Metrics

As part of the primary analysis the pain area was automatically extracted from the pain maps and expressed as pixels and an assessor rated laterality (bilateral or unilateral), generalized pain location on and around the knee, and symmetry. The general location of pain for each pain map was rated as presenting with pain restricted to the peri- or central patellar region and as a third possibility a mixture of both peri- and central patellar pain. Bilateral pain maps were further assessed as symmetric, a tendency for symmetry (borderline symmetric) or clearly non-symmetrical (asymmetric). Symmetric pain was defined as pain drawings in each limb are mirrored images of each other and to a high extent cover the same anatomical areas. Borderline symmetric was defined as pain drawings in each limb are somewhat similar and to a moderate extent cover some of the same anatomical areas. Non-symmetric or asymmetric pain was defined as pain drawings in each limb are not a mirror image and to a very low extent cover the same anatomical areas, if at all.

As a means to understand and quantify the characteristics in the patterns of variation for each cluster an exploratory analysis of the distal to proximal and medial to lateral variance in pain area was performed. The directions were defined as the variance across all columns or rows, respectively, on the pain map (Fig. 1a). Assessing the row and column variance was calculated using equations (1) and (2), respectively, for the left and right knee separately. The left and right knee was analyzed independently to avoid including white space (non-body regions) when calculating the row variance.

1 $${\mu }_{rr}=\frac{1}{A}\sum _{r\in R}\,{(r-\bar{r})}^{2}$$

2 $${\mu }_{cc}=\frac{1}{A}\sum _{c\in R}\,{(c-\bar{c})}^{2}$$

where *r* and *c* are the row and column coordinates of a pixel. *A* is the area (or number of pixels) of the pain region (*R*).

Based on our previous study^17^ pain may spread from the inferior to the superior patellar areas, therefore the maximum pain location distance was determined to explore the possibility of pain spreading in a particular direction. The maximum distal to proximal distance was calculated in the vertical direction only, with respect to the left or right tuberositas tibiae (patellar tendon insertion point on tibia) for each knee (Fig. 1a). The maximum pain location distance was expressed as a vector in pixels and used to gain an understanding if pain spreads distal to proximal rather than a generalized increase in overall pain area.

### Statistics

Data are presented as mean and standard error of the mean (SEM), unless indicated otherwise and non-normality distributed data are expressed as medians with the 25^th^–75th interquartile range (IQR_25_, IQR_75_). Patient demographics (age, sex), patient reported outcomes (Worse pain, pain duration), and pain map metrics (pain area, distal to proximal variation, medial to lateral variation, maximum distal to proximal distance) were evaluated for normality using Shipiro-Wilk tests within the group and for each cluster. For the group, age was normally distributed whereas symptom duration, worse pain, pain duration, and pain map metrics were highly skewed. For normally distributed data, independent t-tests and analysis of variance (ANOVA) was used to assess mean differences between sex and clusters. Post-hoc comparisons were performed using a Bonferroni correction for multiple comparisons. For non-normally distributed data, non-parametric independent samples median tests were used to compare patient reported outcomes and pain map metrics between clusters. Pearson’s chi-square test or the chi-square test of association was used to explore the relationship between sex, pain location and symmetric pain patterns within and between clusters. Post-hoc analysis involved pairwise comparisons using multiple z-tests with two or more proportions with a Bonferroni correction. Mann-Whitney tests were used to compare differences in pain duration between unilateral and bilateral PFP. Spearman’s correlations were used to assess the strength of the relationship between patient reported outcomes and pain map metrics. All analyses were carried out in Statistical Package for Social Sciences (SPSS; version 22, IBM). Significance was accepted for P-values < 0.05.

## Results

Of the 299 participants, both male and female participants were adequately represented with females representing 57% of the total sample of pain maps and can be individually viewed (Supplemental File 299 Individual PFP drawings.pdf).

### Pain Map Clusters

The top six PCs explained 49.7% of the pain map variation, with PC1 = 28.0%, PC2 = 6.8%, PC3 = 5.1%, PC4 = 4.2%, PC5 = 3.0%, and PC6 = 2.6% and characterize the major differences in pain images across the dataset. Both PC1 and PC2 are distinctly higher and the remaining PCs begin to plateau, thereafter these two PCs were chosen to include in the analyses. PC1 and PC2 can be seen in Fig. 1 and notably PC2 consists of positive and negative values indicating spatially anisotropic (completely opposite) pain maps (Fig. 1c).

**Figure 2:**
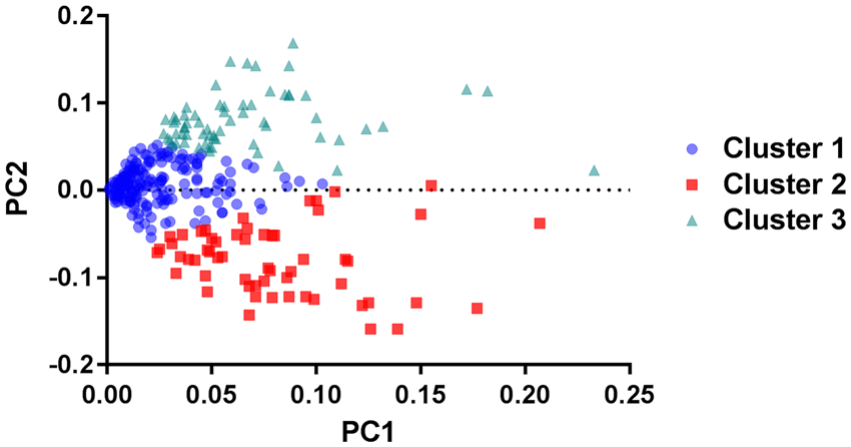
The feature space showing the relationship between principal components 1 and 2 (PC1 and PC2, respectively) and each of the three identified clusters by K-means. Each data point represents a pain drawing.

According to the Elbow method, the recommended number of clusters was K = 4 however the fourth cluster was very small (N = 20). Thus, three clusters were considered to represent the best division and clinical interpretations. The eigenvalues of PC1 and PC2 and the relation to the three identified clusters are shown in Fig. 2.

### Patient demographics and their association with pain clusters

There was no particular association of male or females to one of the three clusters (*Χ*^2^(2) = 0.143, p = 0.931). For the total sample, the age of the female (19.1 ± 8.2 yrs. 95% CI [17.9, 20.2]) as compared to male participants (23.1 ± 8.2 yrs. 95% CI [21.6, 24.5]) was lower (t(298) = −4.11, p = 0.001, d = −3.93). No difference in age was found between the three identified clusters (Table 1; F(2, 296) = 0.668, p = 0.514).

**Table 1:** A summary of patient demographics and patient reported outcomes across the cohort and for the Anchor, Hook and Ovate pain distribution patterns.

	Female	Bilateral	Age (yrs.) *M* ± *SD*	Pain Duration (Months)	Worse Pain*	Pain Area (Pixels)
Overall (299)	173	204	20.8 ± 8.3	24.0 (12.0–48.0)	5.0 (3.3–7.0)	3685 (1834–6293)
Anchor (178)	103	100	20.4 ± 8.5	19.0 (10.0–48.0)	6.0 (3.5–7.0)	2128 (1142–3594)
Hook (64)	36	56	21.7 ± 8.1	25.0 (10.5–60.0)	5.0 (4.0–7.0)	6038 (4123–8547)
Ovate (57)	23	48	21.1 ± 8.1	36.0 (12.0–57.0)	5.0 (3.0–7.0)	7634 (4914–11274)

Data are expressed as means and standard deviations (SD) and medians with the 25th–75th interquartile range (IQR_25_, IQR_75_). *Worse Pain over the last 24 hours are based on 285 samples, with Anchor = 167, Hook = 63 and Ovate = 55.

### Location and distribution of pain

**Figure 3:**
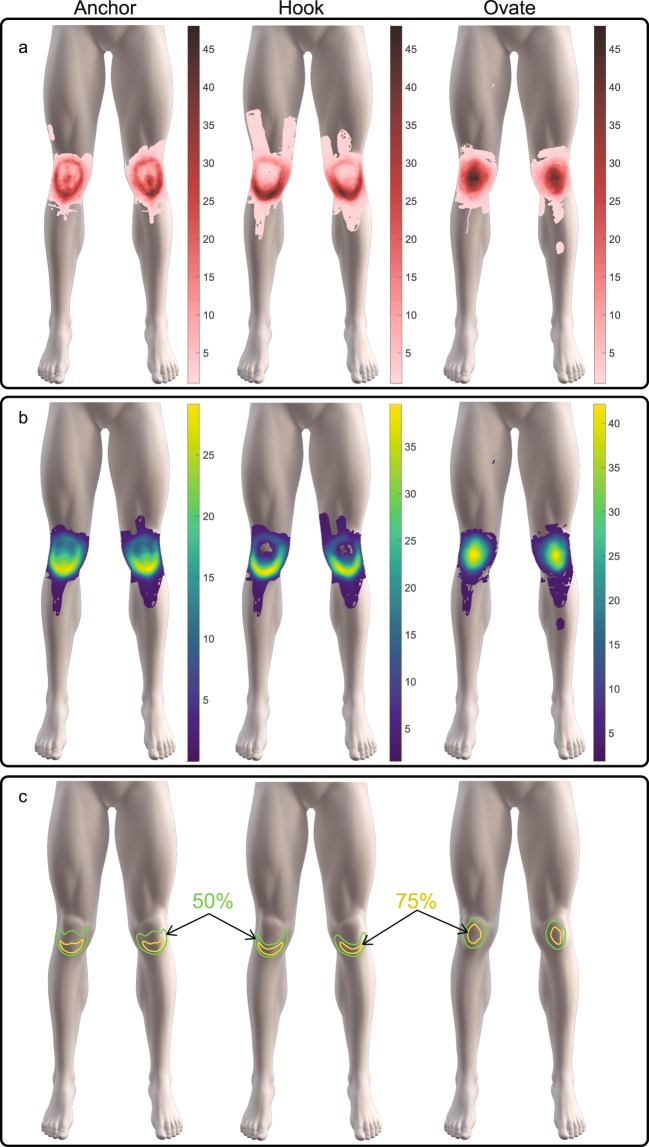
The spatial distributions and superimposed overlays of (**a**) the original pain drawings with common color scales for comparison and (**b**) the reconstructed pain maps based on the top two principal components with color scales for highlighting the common regions. In (**c**) the data-driven boundary plots fare are illustrated rom the reconstructed pain maps outlining the locations in the in which 50% and 75% of all individuals within the Anchor, Hook and Ovate pain distributions have in common.

The original pain maps associated with each cluster are superimposed and shown in Fig. 3 together with the reconstructed pain map using PC1 and PC2. The reconstructed pain maps show the main variations in the pain pattern distributions by displaying the different frequencies of pain distribution on and around the knee across the three clusters. Boundary plots showing common locations for 50% and 75% of all individuals are presented for each cluster in Fig. 3. These boundary plots depict a clear contour of the shape and morphology of the emerging pain distribution patterns.

For Cluster 1, the boundary plots resemble an *Anchor* shape with 75% and 50% of all individuals presenting with pain in peripatellar and central patella regions, respectively (Fig. 3). For Cluster 2, the pain distribution resembles a *Hook* shape emerged for Cluster 2 with 75% and 50% of all individuals presented with pain restricted to the peripatellar regions. For cluster 3, the pain distribution was *Ovate* with 50% and 75%% of all individuals presenting with pain in central patellar region, respectively.

The proportion of individuals with PFP restricted to the peripatellar, central patellar, and peri- and central patellar (mix) regions differed within the cohort (*Χ*^2^(2) = 92.756, p < 0.001). Specifically, a higher proportion of individuals presented with PFP restricted to either the peripatellar (n = 158, 52.8%) or a mixture of both the peripatellar and central patellar regions (n = 116, 38.8%) whereas a low proportion presented with pain restricted to the central patellar region (n = 25, 8.4%).

The proportion of individuals presenting with peripatellar, central patellar or mixture of peripatellar and central patellar pain also differed within and between the Anchor, Hook and Ovate clusters (*Χ*^2^(2) = 83.047, p < 0.001). As summarized in Table 2 pain maps restricted to the peripatellar structures tends to associate with the Anchor cluster (p = 0.0048). A distinguishing feature for the Hook cluster is the absence of individuals with pain in the central patellar region (p < 0.001). In contrast, a very low proportion of individuals with PFP restricted to the peripatellar structures associated with the Ovate cluster (p < 0.001) and instead a mix of both peripatellar and central patellar pain describe this group (p < 0.001).

**Table 2:** A summary of the proportion of individuals with PFP restricted to the peripatellar, central patellar, and a mix of both peripatellar and central patellar structures within and between the Anchor, Hook and Ovate clusters.

Within Cluster	Peripatellar	Central patellar	Mix (Peri- and Central)	Total
Overall	High (52.8%)	Low (8.4%)	High (38.8%)	100%
Anchor	High (59.6%)	Expected (7.9%)	Low (32.6%)	100%
Hook	High (79.7%)	Low (0.0%)	Low (20.3%)	100%
Ovate	Low (1.8%)	Low (19.3%)	High (78.9%)	100%
**Between Cluster**	**Anchor**	**Hook**	**Ovate**	
Peripatellar	High (67.2%)	High (32.3%)	Low (0.63%)	100%
Central patellar	Expected (56.0%)	Low (0.0%)	High (44.0%)	100%
Peripatellar and central patellar	Expected (50.0%)	Low (11.21%)	High (38.79%)	100%

High and low indicates significantly greater or lower than the expected proportion, respectively, as assessed with a post hoc analysis involving pairwise comparisons using multiple z-tests with a Bonferroni correction. Statistical significance was accepted at p < 0.005.

### Pain Duration

For the cohort, pain duration ranged from 2–300 months and differed between the Anchor, Hook and Ovate clusters (Table 1, *Χ*^2^(2) = 8.653, p = 0.013). Pain duration was less for individuals presenting with an Anchor as compared to the Ovate distribution (p = 0.04) and approached significance between the Anchor and Hook distribution (p = 0.08). As expected a positive correlation between pain duration and age was evident (r_s_(299) = 0.356, p < 0.001) present in all three clusters. A sub-group analysis showed pain duration was less for adolescents (18.0 months, IQR = 9.0–30.0) as compared to adults (36.0, IQR = 15.5–69.0) (U = 7.278, p < 0.001).

#### Pain intensity

Worse pain experienced over the last 24 hours ranged between 0–10 for the whole group and no differences were evident between the Anchor, Hook and Ovate clusters (*Χ*^2^(2) = 0.269, p = 0.874).

### Pain Area

**Figure 4:**
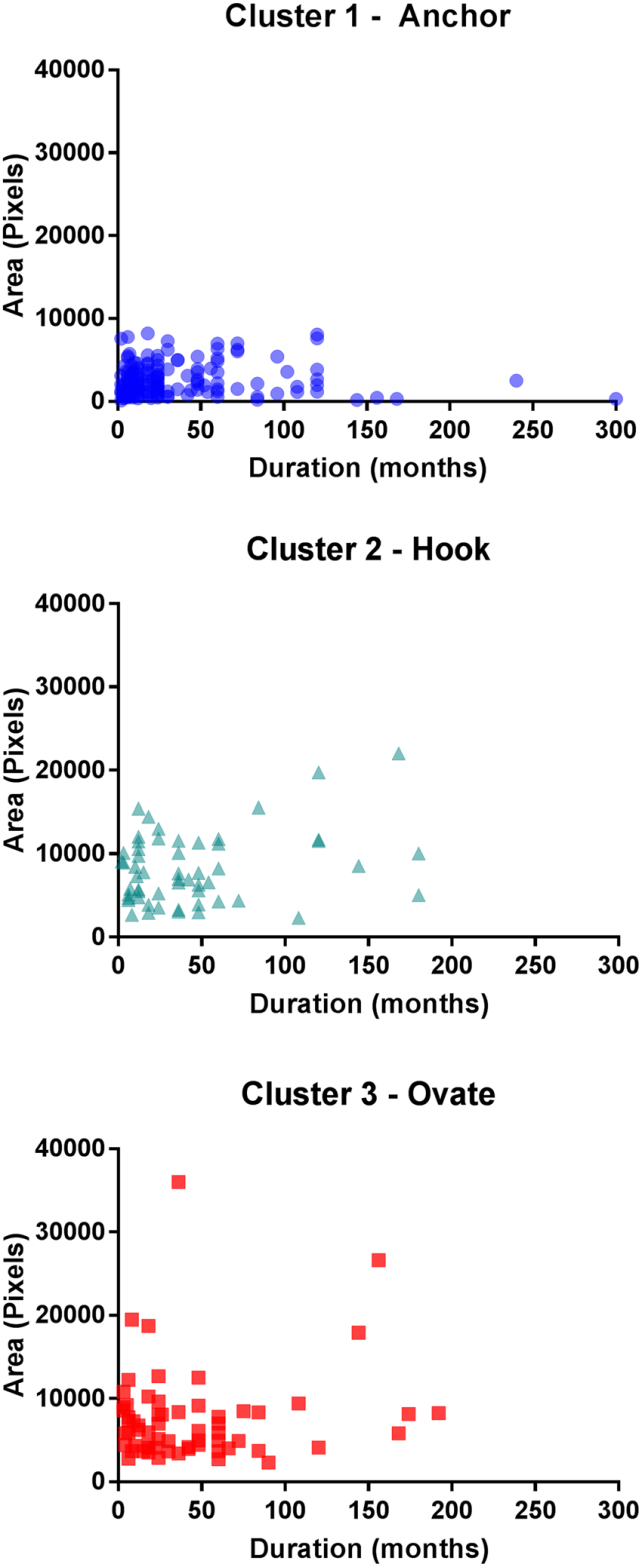
The overall pain area (left and right knee) and the duration of pain symptoms showing no significant relationship with each of the Anchor, Hook and Ovate pain distribution patterns.

Total pain area differed between the Anchor, Hook and Ovate clusters (*Χ*^2^(2) = 113.033, p < 0.001). Post hoc analysis revealed that total pain area for the Anchor was lower than the Hook (p < 0.001) and Ovate (p < 0.001) distributions. A weak positive correlation was found for pain duration versus total pain area (rs(299) = 0.138, p = 0.017), but this was not maintained within the Anchor, Hook and Ovate distributions (Fig. 4a–c).

#### Exploratory Analysis of Pain Area

**Figure 5:**
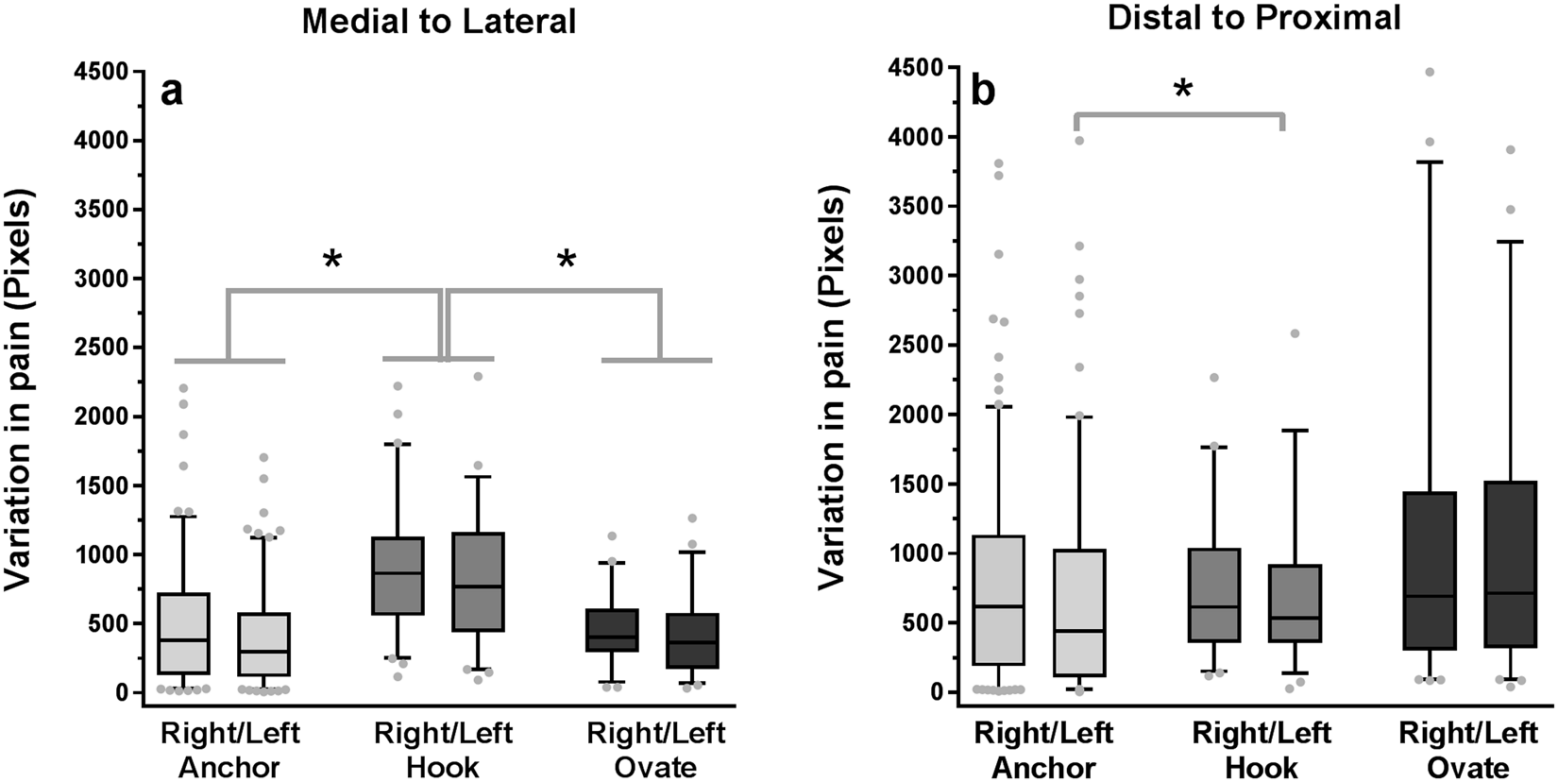
The variance in pain distribution as viewed from (**a**) medial to lateral and (**b**) distal to proximal within the Anchor, Hook and Ovate pain distribution pattern. * indicates a significant difference (p < 0.001) between clusters or left or right knee across clusters.

The medial to lateral variance in pain area differed between the Anchor, Hook and Ovate clusters for the right (*Χ*^2^(2) = 30.253, p < 0.001) and left knee (*Χ*^2^(2) = 27.132, p < 0.001). Post hoc analysis revealed that the medial to lateral variance in pain area for the Hook was greater than the Anchor (p < 0.001) and Ovate (p < 0.001) clusters for the left and right knees, as shown in Fig. 5a.

The distal to proximal variance in pain area differed between the Anchor, Hook and Ovate distributions for the left (*Χ*^2^(2) = 10.576, p = 0.005) but not for the right (*Χ*^2^(2) = 0.743, p = 0.690). Post hoc analysis revealed that the variation in pain area from distal to proximal was lower for the Anchor as compared to the Hook (p = 0.005) distribution as shown in Fig. 5b.

The maximum pain location distance, in the distal to proximal direction, with respect to the tibial tuberosity (patellar tendon insertion point on tibia) was not correlated with the duration of pain symptoms and no differences between the clusters or laterality (unilateral vs. bilateral) evident.

### Bilateral versus unilateral pain

For the cohort a higher proportion of individuals presented with bilateral PFP (68.2%) and 14.4% and 17.4% presented with pain on their right or left knee (*Χ*^2^(2) = 19.042, p < 0.001). A sub-group analysis on adolescents (<20 years, n = 142) however also revealed a higher proportion of bilateral PFP (n = 97, 68.3%, *Χ*^2^(2) = 19.042, p < 0.001). According to the pain maps, those with bilateral pain demonstrated a higher proportion of peripatellar (n = 103, 50.5%) and a mixture of peri- and central patellar pain (n = 86, 42.2%) and a lower proportion of central patellar pain (n = 15, 7.4%, *Χ*^2^(2) = 64.088, p < 0.001).

Pain duration was greater for those presenting with bilateral (24.0 months, IQR = 12.0–60.0) compared to unilateral PFP (18.0 months, IQR = 6.0–36.0, U = 12.244, p < 0.001). There were differences in the proportion of bilateral versus unilateral pain within the Anchor (56.2.0% versus 43.8.2%), Hook (87.5% versus 12.5%), and Ovate clusters (84.2% versus 15.8%, *Χ*^2^(2) = 29.601, p < 0.001).

When assessing only those with bilateral pain the differences in pain duration between the three clusters did not hold (*Χ*^2^(2) = 3.072, p < 0.215). However, the total pain area was still lower for the Anchor as compared to the Hook and Patella when assessing only those with bilateral (*Χ*^2^(2) = 62.979, p < 0.001, post hoc, p < 0.001) and unilateral pain (*Χ*^2^(2) = 21.146, p < 0.001; post hoc, p < 0.009).

### Pain symmetry in patients with bilateral pain

**Figure 6:**
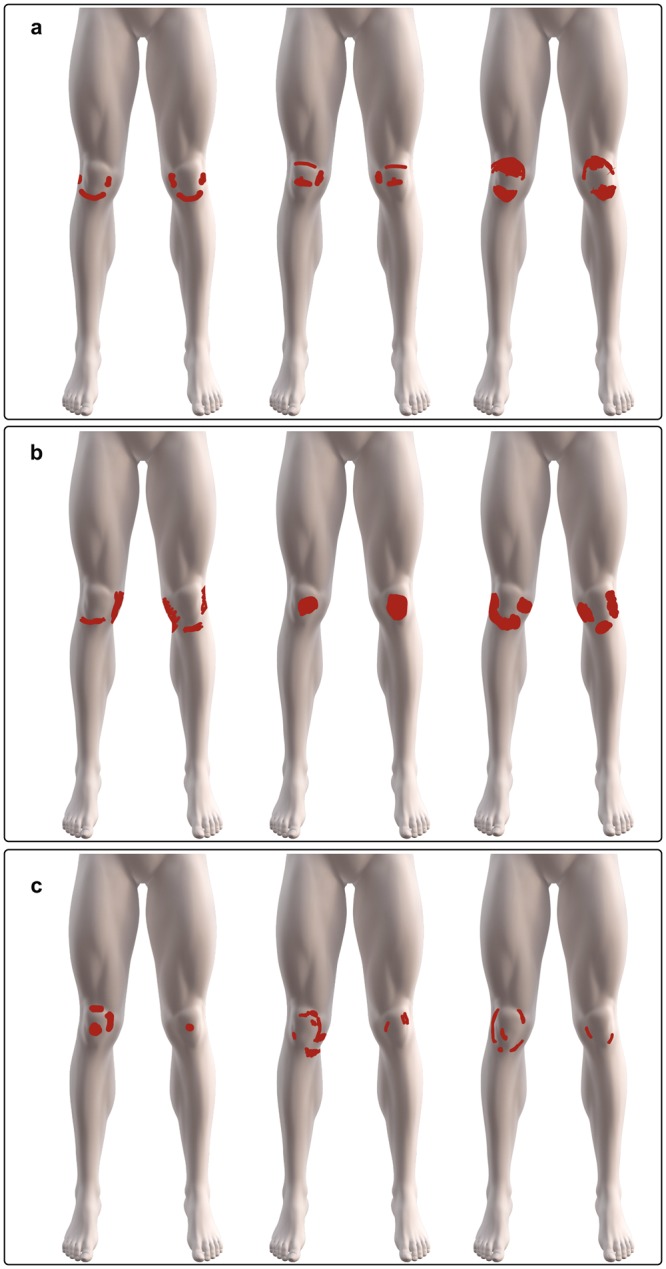
Examples of bilateral PFP drawings showing (**a**) symmetrical (**b**) borderline symmetrical (**c**) and non-symmetric patterns of pain location between the left and right knee.

For those with bilateral pain, symmetric pain was evident in more than half (n = 115, 56.4%) whereas borderline symmetric (n = 60, 29.4%) and non-symmetric (n = 29, 14.2%) pain patterns were less common, (*Χ*^2^(2) = 55.794, p < 0.001). Symmetric, borderline symmetric, and asymmetric PFP drawings were equally distributed across the Anchor, Hook and Ovate distributions (*Χ*^2^(2) = 7.470, p = 0.113) and examples of each are shown in Fig. 6.

## Discussion

This study is the first to identify three distinct patterns of variation in the spatial distribution of knee pain as acquired using pain drawings in patients reporting PFP. The primary patterns of variation resemble an Anchor, Hook and Ovate shape, respectively, on and/or around the patella. Despite the high prevalence of PFP, little is known about the underlying PFP pathology and it is tempting to speculate if these findings hint at specific structures being affected. Our previous study documented that PFP is generally peripatellar or central patellar pain^17^ but the present results uncovered three distinct patterns that may be related to the underlying pathology. With the exception of pain duration and area, these three patterns were independent of sex, age, and pain intensity. Patients with bilateral pain had a longer duration of pain and more often belonged to the Hook and Ovate pain distributions. Two dominant features observed across the entire cohort were bilateral pain and pain restricted to the peripatellar region.

Considering the spatial location of these pain distributions about the knee the Anchor shape may involve the peripatellar and central patellar structures, such as the patellar tendon^26^. In contrast, the Hook distribution can be described as pain located primarily in the peripatellar structures such as the fat-pad^27^. The Ovate distribution depicts central patellar pain and may reflect pain stemming from multiples sources including the subcutaneous peripatellar bursa or from deeper structures behind the patella, such as the synovial membrane^28^. In this study, over half of the individuals belonged to the Anchor distribution cluster and this cluster is characterized by (1) lower overall pain area, (2) a higher proportion of pain restricted to the peripatellar structures, and (3) a lesser duration of pain symptoms. The Anchor distribution may consist of a more localized presentation of pain that involves the inferior part of the patella bone and/or Hoffas fat pad^26^ as supported by the relatively smaller pain area and lower medial to lateral variance.

Previously we hypothesized that the trajectory of pain with increasing pain duration may spread from a U to an O shape on and around the patella^16^ and indicate symptom progression. We did not find evidence in support of this hypothesized trajectory of pain spread with increasing duration of pain as the duration of pain did not correlate with the distance in maximum pain location relative to the patellar tendon insertion point. Although these findings are based on cross-sectional data they suggest pain spread may not necessarily have a preferred direction as the duration of pain increases and further prospective assessments are needed to understand such characteristics.

Conceivably one pain distribution pattern could change, expand or transition to another. Entertaining this notion, the Anchor distribution which has the shortest duration of pain symptoms and lower area of pain relative to the Hook and Ovate would be the most likely candidate. The Hook distribution is characterized by pain restricted to peripatellar region and thus only individuals from the Anchor group with pain restricted in this region could expand in the medial to lateral direction with extended symptom duration. This possibility would be supported by the higher prevalence of peripatellar pain in the Anchor cluster and a higher medial to lateral variance in the Hook distribution. The Ovate distribution is rather distinct and principally expresses pain located more centrally (central patellar) with little to no pain present in the inferior peripatellar region. Given that the overall pain area for the whole cohort is only weakly correlated with increasing duration of pain symptoms there is little support for the notion that one pain distribution pattern expands and transitions into another. Instead the identified pain distributions patterns are relatively unique.

Whether pain distribution patterns, site and overall area in PFP present differently when the duration of pain symptoms are relatively acute (less than 6 months) or when the patient is very young (e.g. 10–14 years) is uncertain. What is clear is that pain location in patients experiencing PFP appears to be unrelated to the intensity of pain. In the healthy knee, the anterior synovial tissues, fat pad, and capsule are extremely sensitive to mechanical stimuli^29^. The most sensitive sites or locations to mechanical pressure however differ about the knee between healthy controls and those with severely painful knee osteoarthritis (OA)^29^. Thus, pain sensitivity is not necessarily equally enhanced at all locations but may be disproportionally altered depending on the underlying driving mechanisms. To expand on this notion, the structures capable of driving PFP may differ between individuals and may be reflected in the pain distributions patterns as pain duration progresses from acute to chronic. Mapping and tracking pain-related symptoms at onset would shed light on the progression and trajectories of pain spread as studies on longitudinal changes in knee pain localization and area have yet to occur. How pain initially presents and spreads with symptom duration is critical knowledge that is lacking. Cross-sectional studies revealed that localized, regional or diffuse knee pain in older adults with knee OA differentially associate with risk factors and progression of knee OA symptoms^30^. Greater areas (diffuse anterior knee pain compared to local or regional pain) is more strongly associated with older age, higher BMI and hand OA^30^. To date there is limited knowledge about the progression of knee pain, including but not limited to PFP and OA, and mapping the progression and transitions characteristics of pain distribution could lead to valuable prognostic markers.

One characteristic which may represent a progression of PFP symptoms is the transition from unilateral to bilateral knee pain. In this study, bilateral pain was associated with a longer duration of pain symptoms. Further, a higher proportion of bilateral pain was evident for the Hook and Ovate pain distribution distributions. Relative to the Anchor distribution, bilateral pain may be the explanatory factor for why the Hook and Ovate pain pattern distributions are characterized by a longer duration of pain symptoms. In the current study, even adolescents with PFP demonstrated a similar proportion of unilateral and bilateral pain, so age itself is not a defining characteristic. Whether individuals fitting a PFP diagnosis commonly manifest as unilateral pain and with extended duration of symptoms progress to a bilateral pain pattern remains to be confirmed. To date, bilateral pain in symptomatic knee osteoarthritis^31^ and low back pain^11^ are known to associate with more severe conditions. Thus, bilateral pain is potentially a marker of symptom severity for a number of painful conditions. A transition from unilateral to bilateral pain fits with the theoretical model of more widespread pain with increasing pain duration^19^. For example, widespread pain, as documented by pain mapping, in patients with knee osteoarthritis associate with increased severity and central sensitization^32^.

The current study validates our earlier findings and reveals a high proportion of individuals with bilateral pain demonstrate a symmetric pain pattern^17^. In this current study, pain in the left and right knee appear as mirror image in over 50% of those with bilateral pain and an additional 30% show a tendency for symmetry. Bilateral and symmetrical pain was similarly present in all clusters suggesting that symmetry occurs irrespective of pain distribution or location. The unambiguous high degrees of symmetry or ‘mirror image’ pain is a newly discovered pain location description^17^ and in accord to this study an evidently clear dominant characteristic in patients presenting with PFP. In the past, symmetric pain or symptom patterns were generally defined as appearing in the same bodily region^10,33,34^, despite this few studies have attempted to explain the phenomenon. Egloff and colleagues (2012), collected 62 pain maps from patients recruited from a psychology department including but not limited to fibromyalgia, chronic tension headache, chronic low back, functional abdominal pain and an orthopedic department including traumatic pain of the upper and lower extremities identified symmetry as one of three criteria for differentiating between somatoform-functional pain and somatic-nociceptive pain^10^. Somatoform-functional pain is considered a response to psychosocial stress and somatic-nociceptive pain from injury to the muscles, joints, tendon and bones. Egloff and colleagues (2012) suggested that symmetrical pain may stem from generalized hyperalgesia or sensitization of the musculoskeletal system^10^ whereas, Stimpson and colleagues (2006) who assessed pain distribution in Persian Gulf War veterans suggested a more ‘systemic type of pain’ as an injury is likely to occur on one side of the body^34^. Indeed, a most recent study found evidence of facilitated peripheral and central pain mechanisms in patients experiencing PFP^28^. An alternative explanation to consider is that symmetric pain reflects an underlying structural pathology leading to an altered biomechanical loading of both knees^35^. What remains unknown is whether other factors such as physical function or bone and soft tissue integrity demonstrate symmetric scores in both knees or if the phenomena has a more central or psychologic origin. A limitation of this study is the subjective classification of symmetric and borderline symmetric patterns. Differences in opinions between clinicians and researchers regarding factors to discriminate symmetric and borderline symmetric are likely and remains an area in need of improvement. In support of this view, our previous study revealed that subjective assessment of symmetry was surprisingly stricter than an automatic algorithmic approach^17^ as the assessors included a deeper understanding of anatomy and physiology. The well-correlated medial to lateral and distal to proximal variance in pain area however support the prevalence of the symmetrical pain patterns across the cohort.

There are likely a high number of variables contributing to the variation in the pain distribution and this can range from the drawing ability of the individual to the source of the individual’s pain. The advantage of using PCA on the PFP maps is the reduction in the number of these variables while capturing most of the variance. While other methods such as independent component analysis, non-negative matrix factorization, or auto-encoders^36^ could be applied to study the variation in the pain maps, PCA has the advantages of having a simple interpretation and efficient computation. Implementing an unsupervised K-means clustering approach using the two most prevalent PCs produced clusters based on the most explanatory variables. The advantage of clustering in a two dimensional space is that the data and resulting clusters can be easily visualized. Further, this study is only concerned with the most common variations in pain distribution observed across the dataset. We note that although multiple clustering algorithms have been proposed, k-means remains a widely used approach due to its simplicity and effectiveness^36^. Subsequently, superimposing the reconstructed pain maps, which are based on the most prevalent PCs, easily highlights the differences in the pain distribution between each cluster.

For many years pain drawings were carried out on paper as part of an anamnesis or documentation of a patient’s pain experience^7,10,11,37,38^. Although paper pain drawings are less modern, it is the quality of the drawing instructions, anatomic detail, and the display size of the body chart that facilitate better communication of pertinent information^3^. Moreover, digital pain drawings enable the acquisition and analysis of large amounts of data which can serve as a basis to track, understand, and predict the trajectory of symptoms. Pain drawings lend well to machine learning approaches^39^ and are beginning to show clinical utility on a number of fronts ranging from patients suffering from pain in the knee^17,32^, back^9,11,12^, shoulder^13^ or neck^4^. As an example, pain or discomfort drawings can capture what magnetic imaging (MRI) may miss^9^ as shown in patients with long-standing nerve root symptoms. For the greatest impact, digital pain mapping studies should, in the future, aim for longitudinal and observational study designs to capture the change in pain presentation over time.

In summary, this study identified three distinct PFP distribution patterns that were independent of sex, age, and pain intensity and revealed that bilateral symmetrical pain is a dominant characteristic in patients presenting with PFP. Methodically, this study underscores the advantages of how digital pain drawings can be utilized to deepen our understanding in areas where critical knowledge is lacking, especially in conditions for which pain is a major symptom.

## Electronic supplementary material

Supplementary Dataset 1

## Data Availability

The patient reported data and the datasets extracted from the pain drawings used and/or analyzed during the current study are available from the corresponding author on reasonable request. However, the pain drawings of all PFP patients used in the main analysis of this study are readily available as a Supplemental File 299 Individual PFP drawings.pdf.
