# Supplementary material for: Distinct patterns of variation in the distribution of knee pain
Source: Sci Rep. 2018 Nov 8;8:16522. doi: 10.1038/s41598-018-34950-2 (PMC6224396; doi:10.1038/s41598-018-34950-2)
Supplement: Supplementary file 1 — Supplementary Dataset 1 [file 41598_2018_34950_MOESM1_ESM.pdf]

Distinct patterns of variation in the distribution of knee pain

Shellie A. Boudreau[1,*], Albert Cid Royo[1], Mark Matthews[2,3], Thomas Graven-Nielsen[1], Ernest N. Kamavuako[4], Greg Slabaugh[5], Kristian Thorborg[6], Bill Vicenzino[2] and Michael Skovdal Rathleff[7,8]

[1] Center for Neuroplasticity and Pain (CNAP), Department of Health Science and Technology, Aalborg University, Denmark

[2] The University of Queensland, School of Health and Rehabilitation Sciences, Sports Injuries Rehabilitation and Prevention for Health research unit, CCRE Spine, Brisbane, Australia

[3] Sports and Exercise Sciences Research Institute, School of Sport, Ulster University, Belfast, United Kingdom

[4] Center for Robotics Research (CORE), Department of Informatics, King's College London, United Kingdom

[5] Department of Computer Science, City, University of London, United Kingdom

[6] Sports Orthopedic Research Center - Copenhagen (SORC-C), Department of Orthopaedic Surgery, Copenhagen University Hospital, Amager-Hvidovre, Denmark

[7] Research Unit for General Practice in Aalborg, Department of Clinical Medicine, Aalborg University, Denmark

[8] SMI, Department of Health Science and Technology, Aalborg University, Denmark

Figure 1: All individual pain drawings displayed in random order with 9 drawings per page.

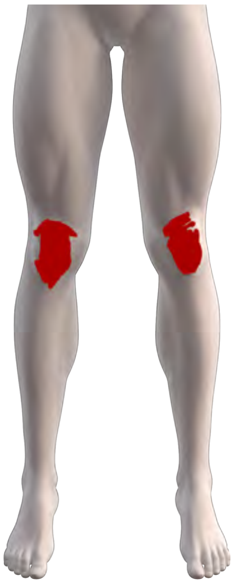

(a) Subject ID: 1

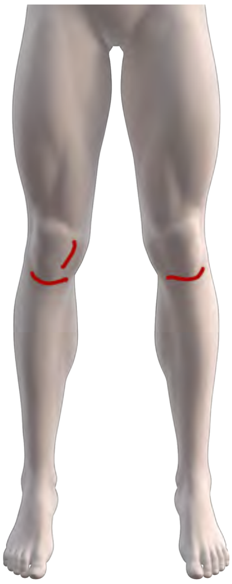

(b) Subject ID: 2

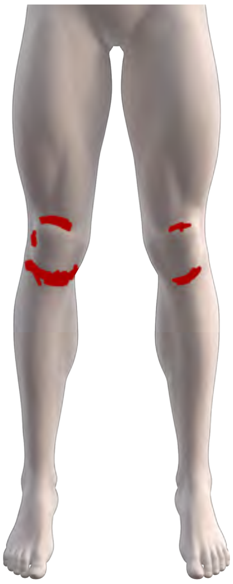

(c) Subject ID: 3

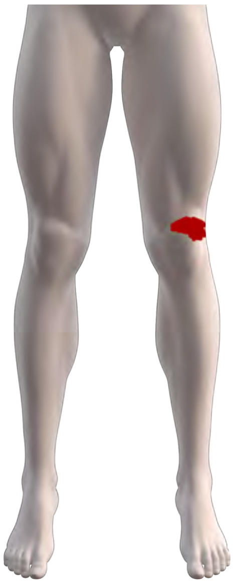

(d) Subject ID: 4

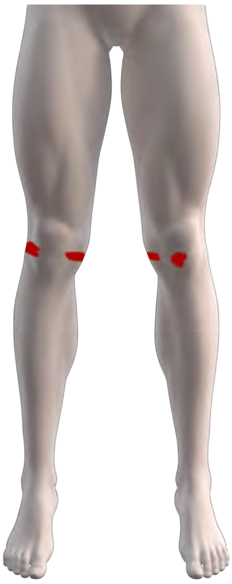

(e) Subject ID: 5

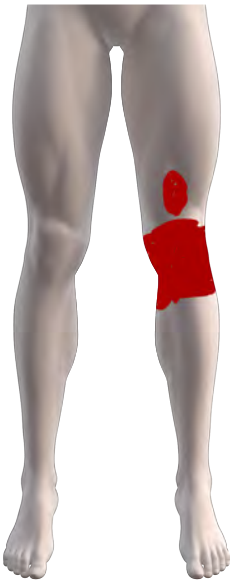

(f) Subject ID: 6

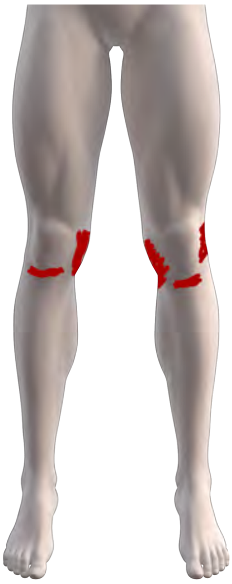

(g) Subject ID: 7

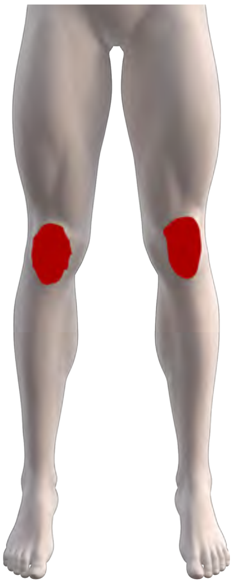

(h) Subject ID: 8

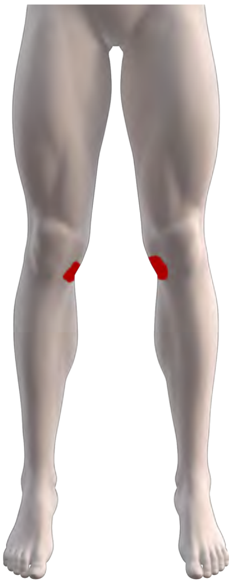

(i) Subject ID: 9

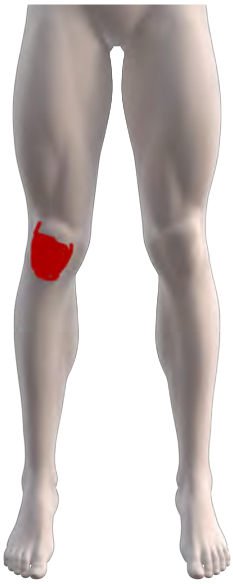

(a) Subject ID: 10

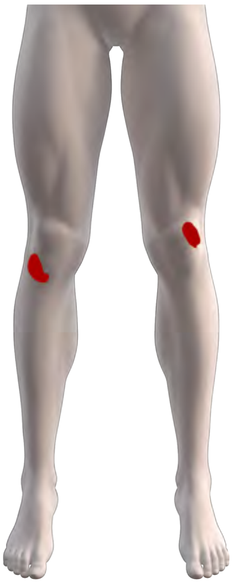

(b) Subject ID: 11

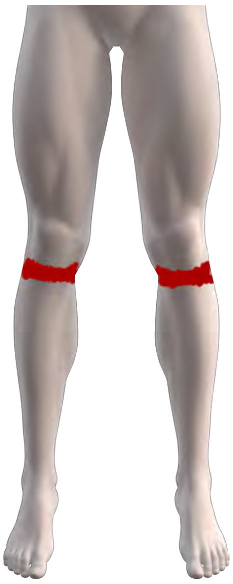

(c) Subject ID: 12

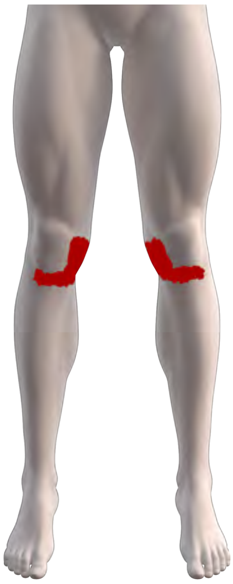

(d) Subject ID: 13

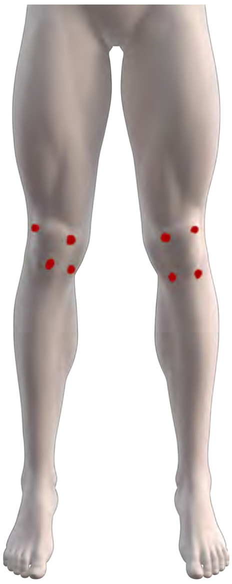

(e) Subject ID: 14

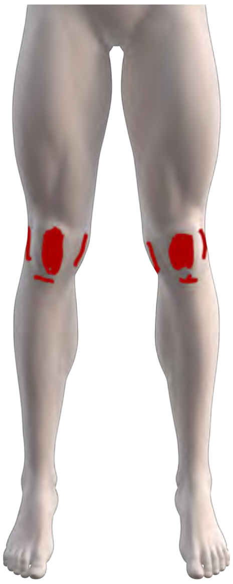

(f) Subject ID: 15

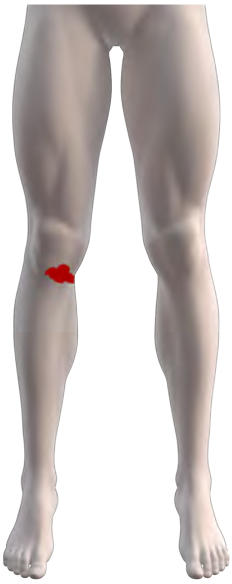

(g) Subject ID: 16

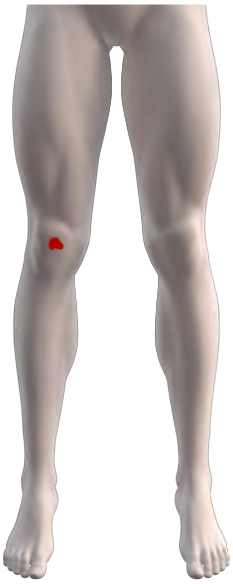

(h) Subject ID: 17

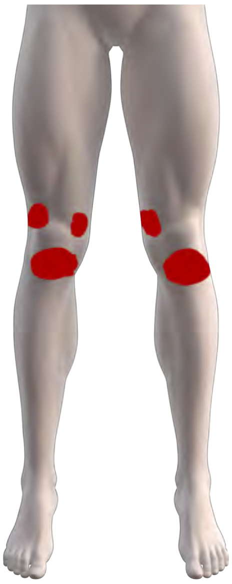

(i) Subject ID: 18

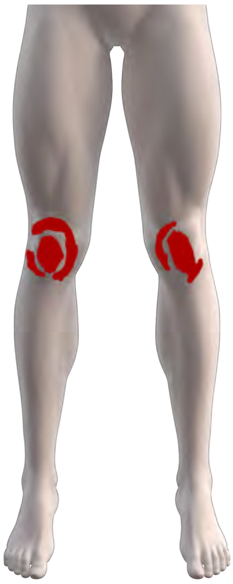

(a) Subject ID: 19

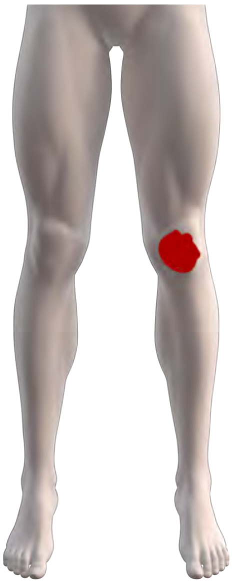

(b) Subject ID: 20

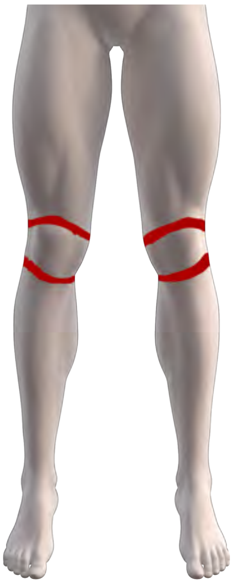

(c) Subject ID: 21

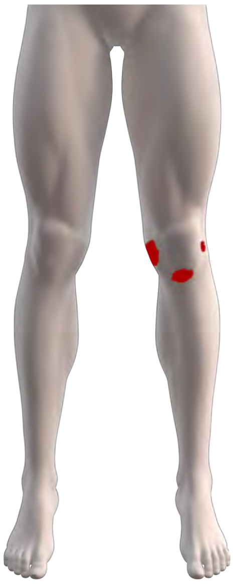

(d) Subject ID: 22

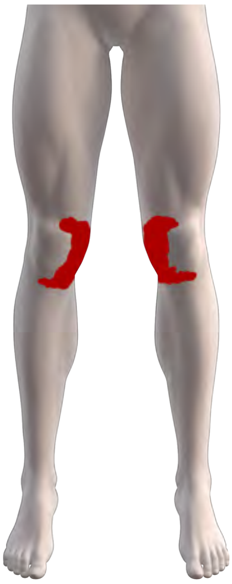

(e) Subject ID: 23

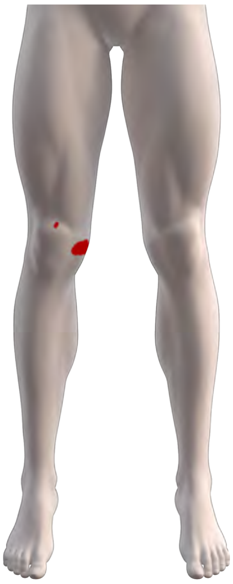

(f) Subject ID: 24

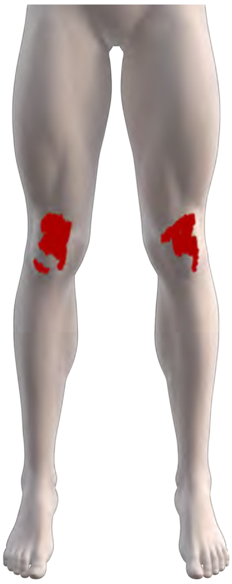

(g) Subject ID: 25

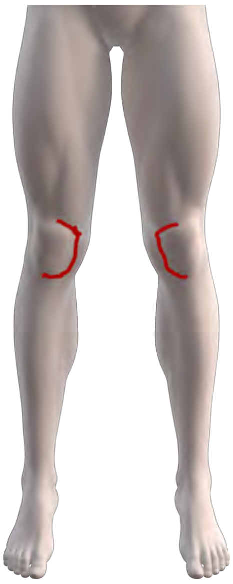

(h) Subject ID: 26

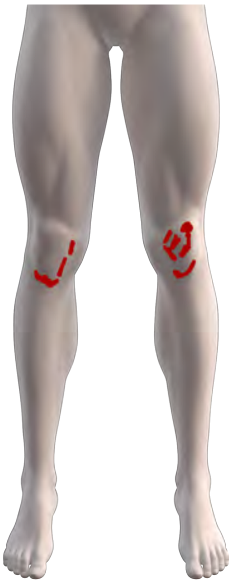

(i) Subject ID: 27

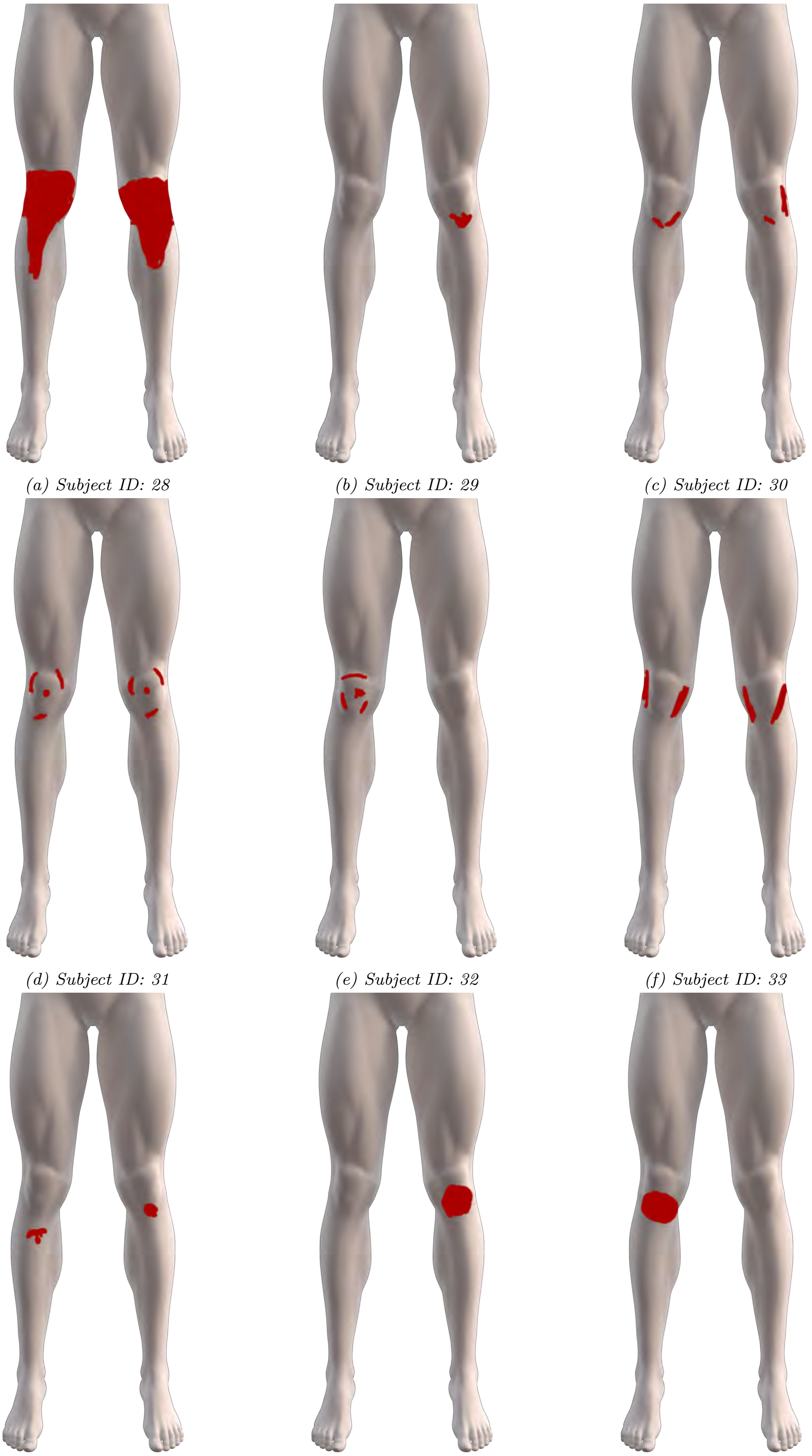

(a) Subject ID: 28

(b) Subject ID: 29

(c) Subject ID: 30

(d) Subject ID: 31

(e) Subject ID: 32

(f) Subject ID: 33

(g) Subject ID: 34

(h) Subject ID: 35

(i) Subject ID: 36

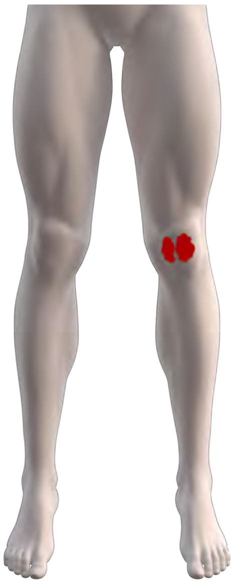

(a) Subject ID: 37

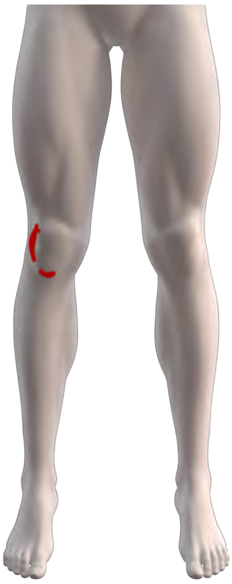

(b) Subject ID: 38

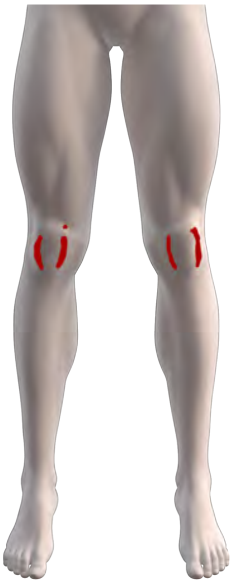

(c) Subject ID: 39

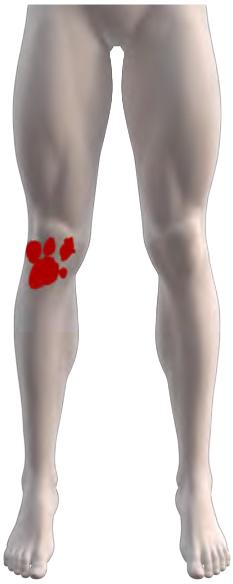

(d) Subject ID: 40

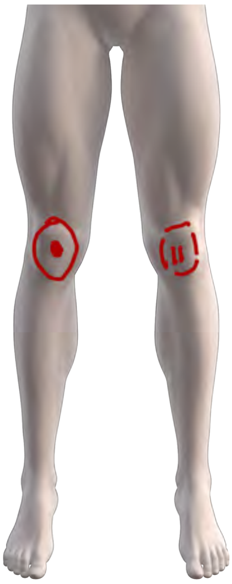

(e) Subject ID: 41

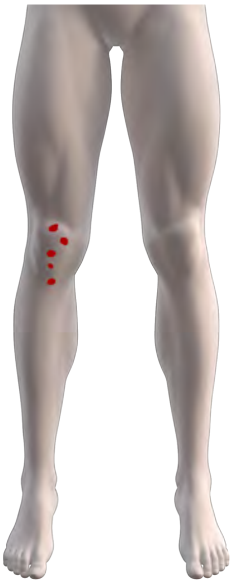

(f) Subject ID: 42

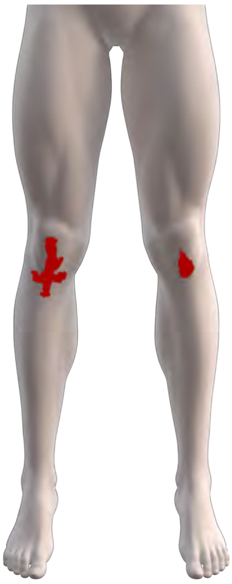

(g) Subject ID: 43

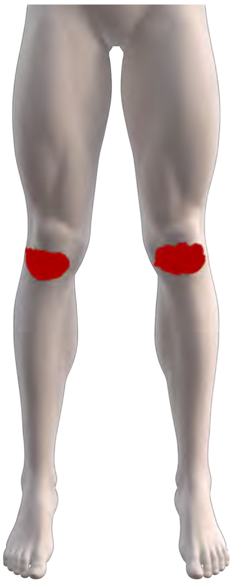

(h) Subject ID: 44

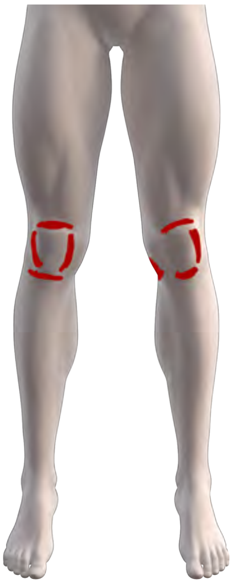

(i) Subject ID: 45

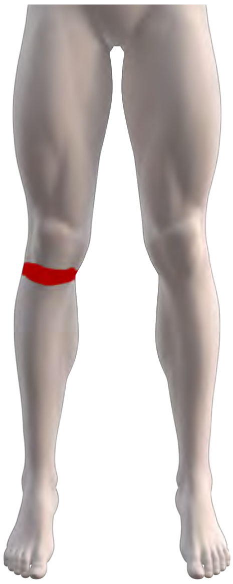

(a) Subject ID: 46

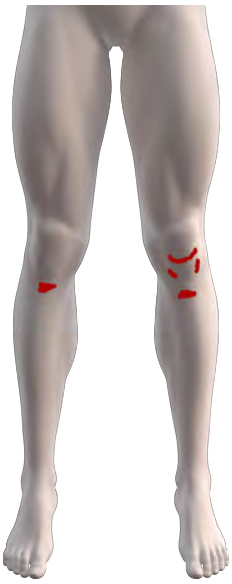

(b) Subject ID: 47

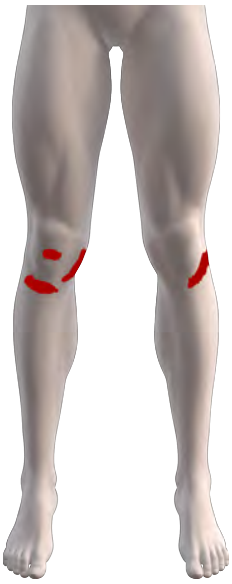

(c) Subject ID: 48

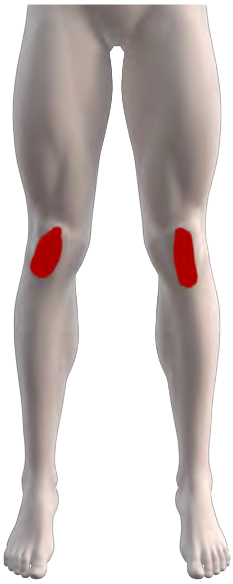

(d) Subject ID: 49

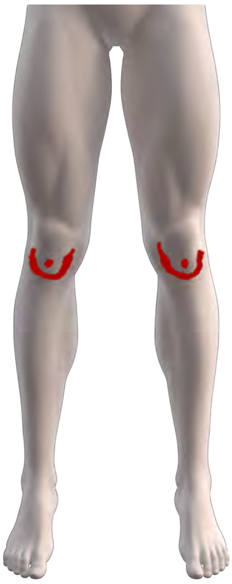

(e) Subject ID: 50

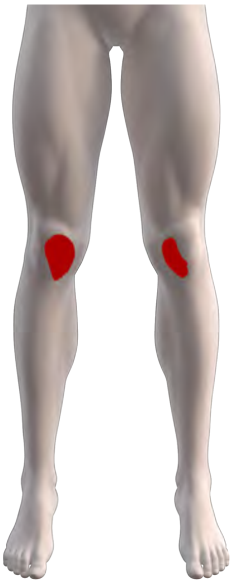

(f) Subject ID: 51

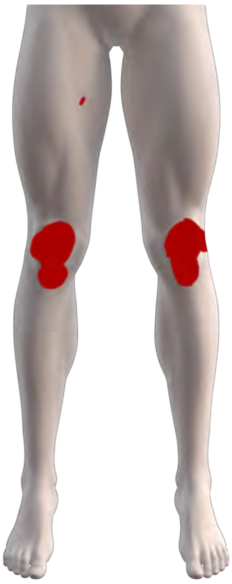

(g) Subject ID: 52

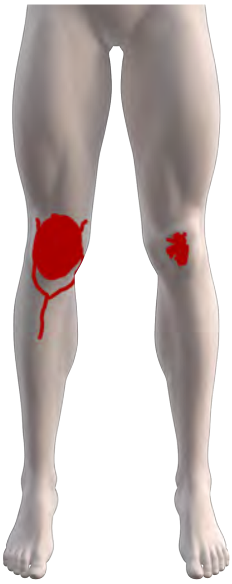

(h) Subject ID: 53

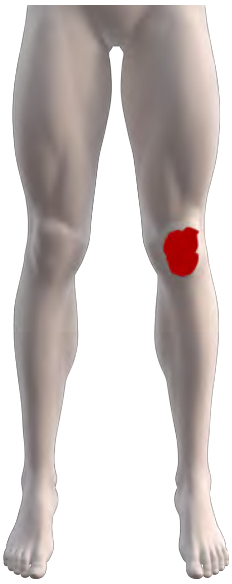

(i) Subject ID: 54

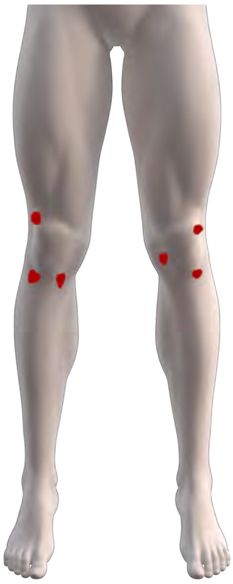
(a) Subject ID: 55

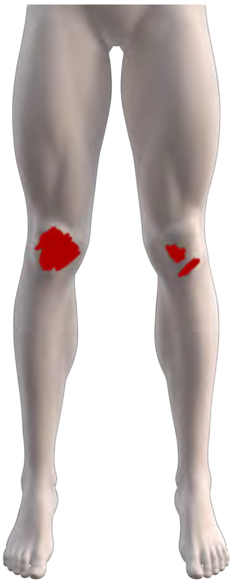
(b) Subject ID: 56

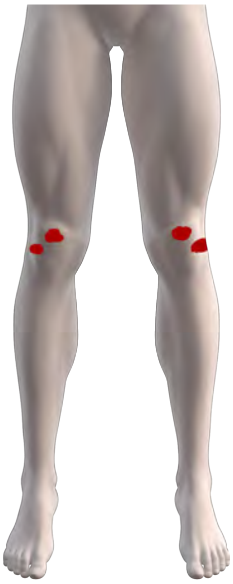
(c) Subject ID: 57

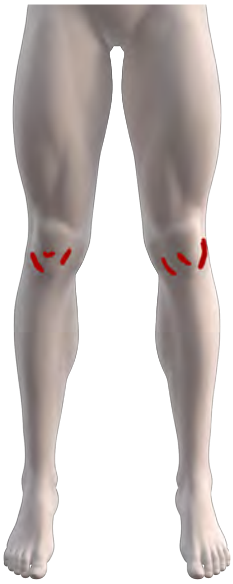
(d) Subject ID: 58

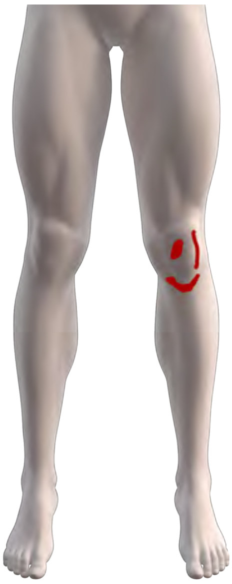
(e) Subject ID: 59

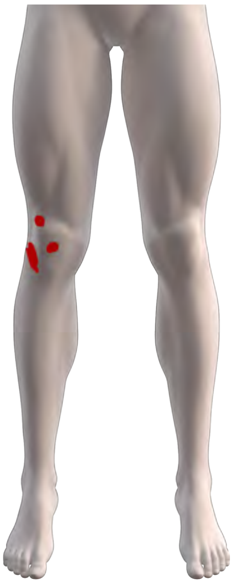
(f) Subject ID: 60

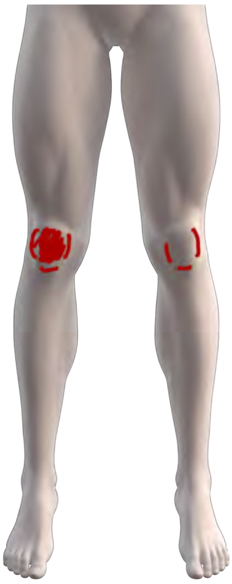
(g) Subject ID: 61

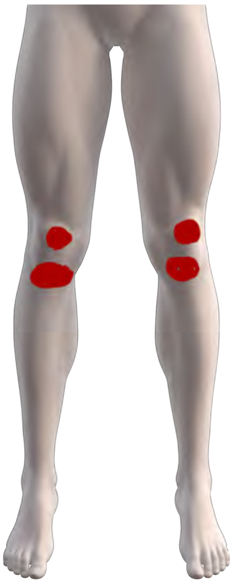
(h) Subject ID: 62

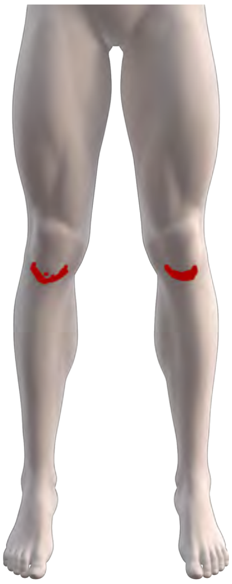
(i) Subject ID: 63

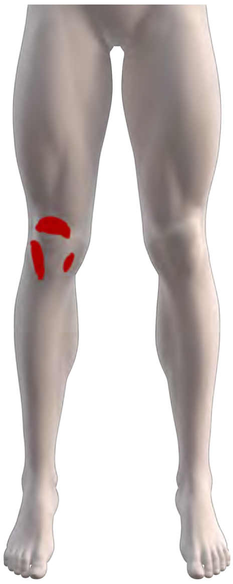

(a) Subject ID: 64

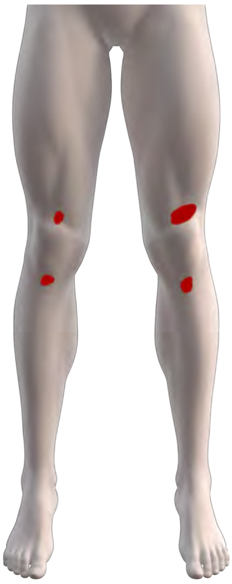

(b) Subject ID: 65

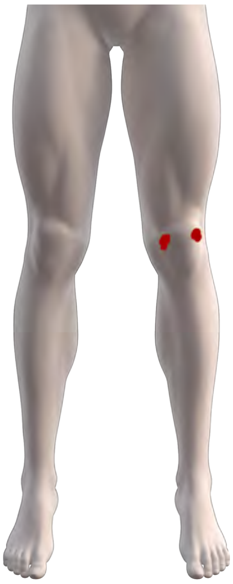

(c) Subject ID: 66

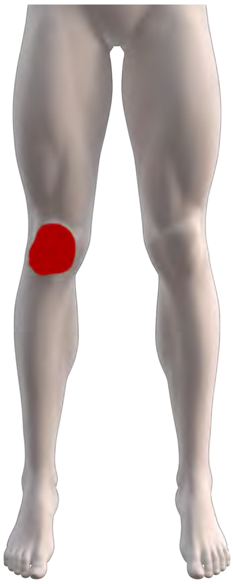

(d) Subject ID: 67

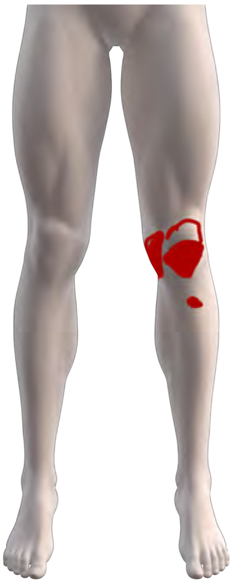

(e) Subject ID: 68

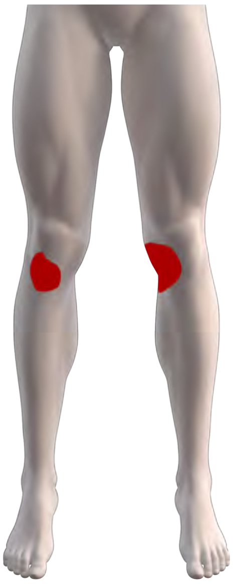

(f) Subject ID: 69

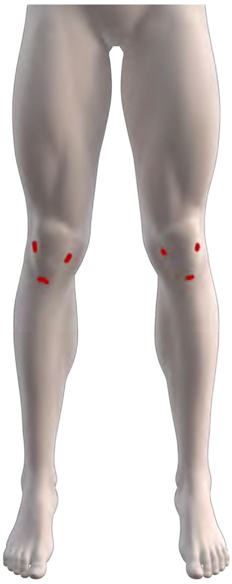

(g) Subject ID: 70

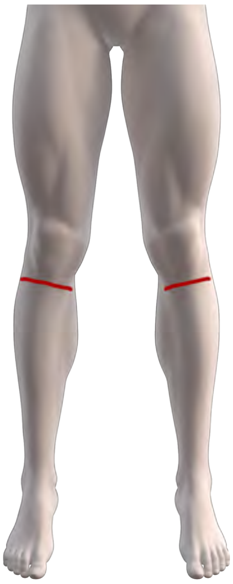

(h) Subject ID: 71

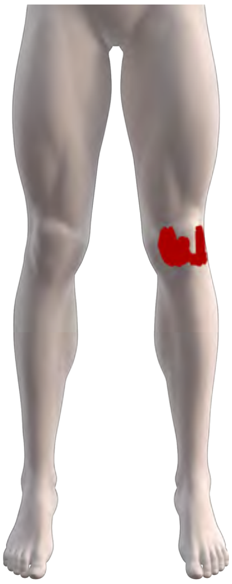

(i) Subject ID: 72

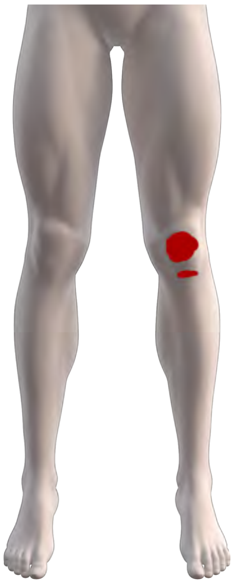

(a) Subject ID: 73

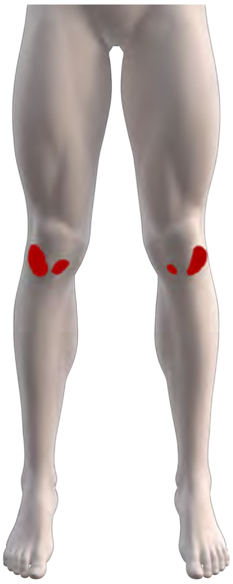

(b) Subject ID: 74

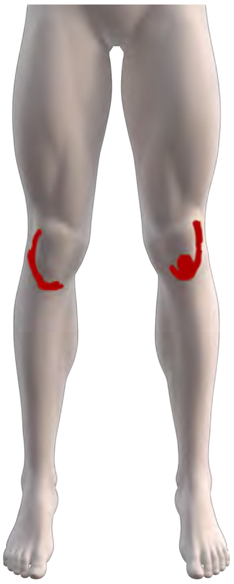

(c) Subject ID: 75

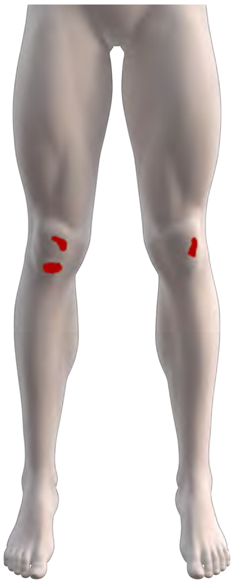

(d) Subject ID: 76

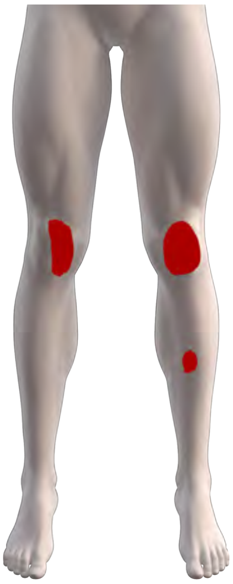

(e) Subject ID: 77

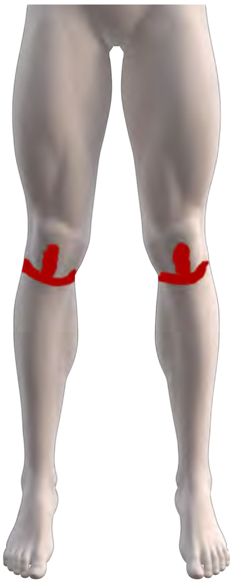

(f) Subject ID: 78

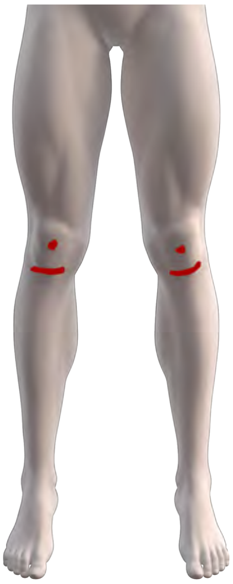

(g) Subject ID: 79

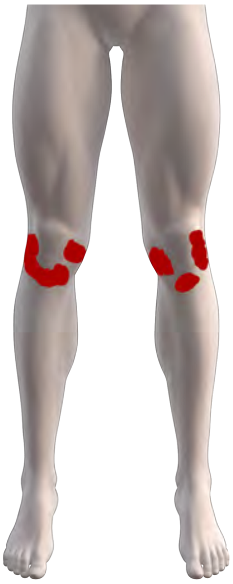

(h) Subject ID: 80

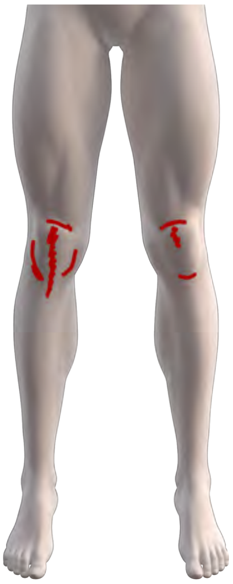

(i) Subject ID: 81

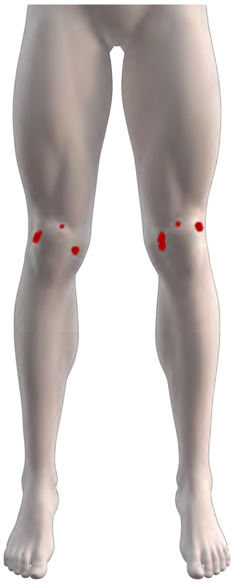

(a) Subject ID: 82

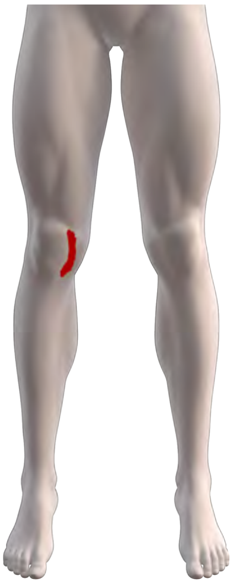

(b) Subject ID: 83

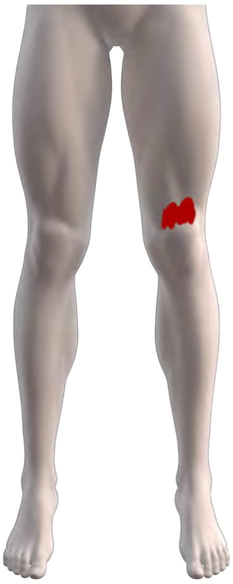

(c) Subject ID: 84

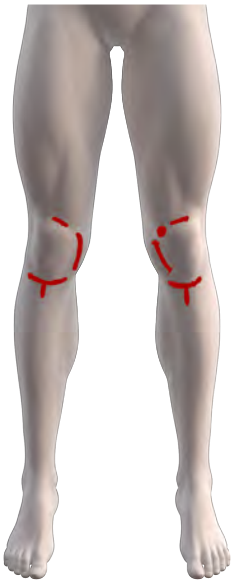

(d) Subject ID: 85

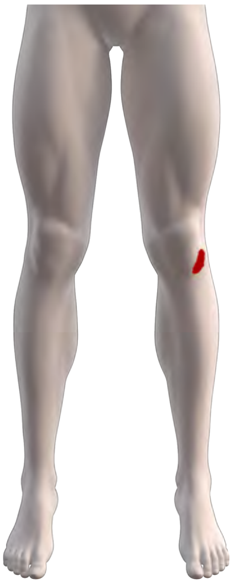

(e) Subject ID: 86

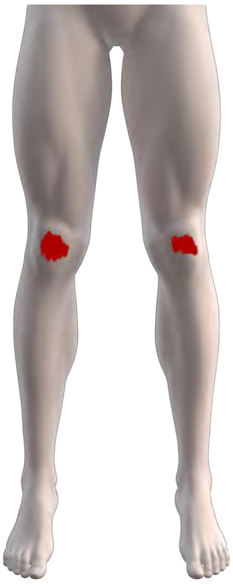

(f) Subject ID: 87

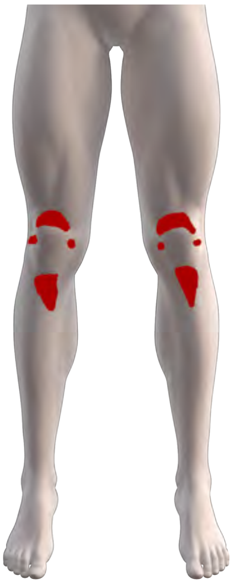

(g) Subject ID: 88

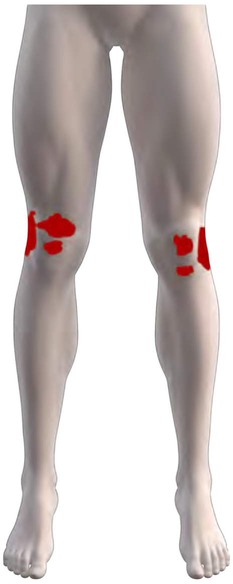

(h) Subject ID: 89

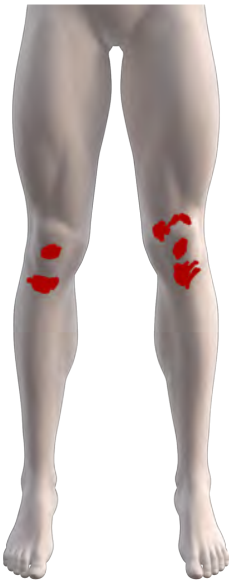

(i) Subject ID: 90

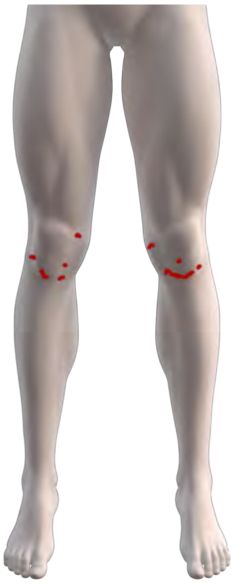

(a) Subject ID: 91

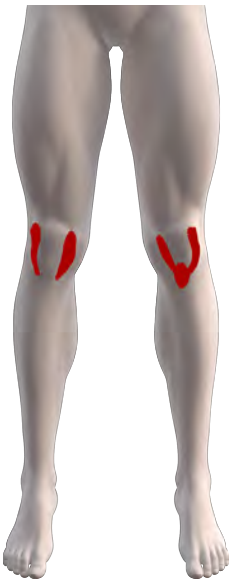

(b) Subject ID: 92

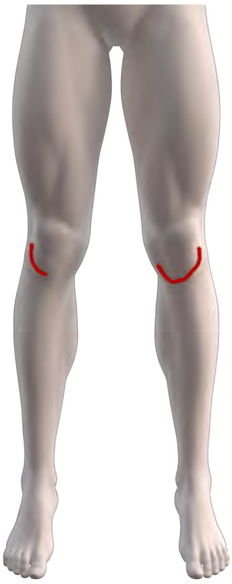

(c) Subject ID: 93

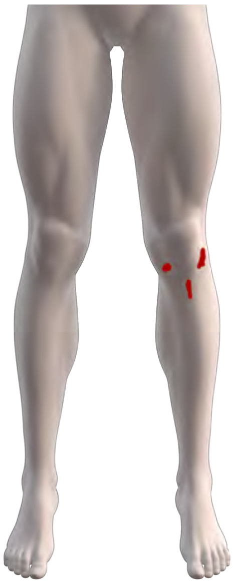

(d) Subject ID: 94

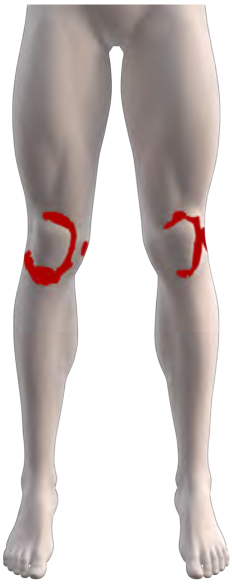

(e) Subject ID: 95

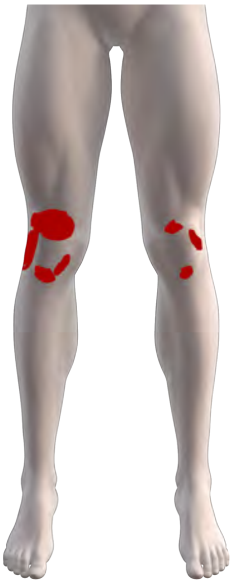

(f) Subject ID: 96

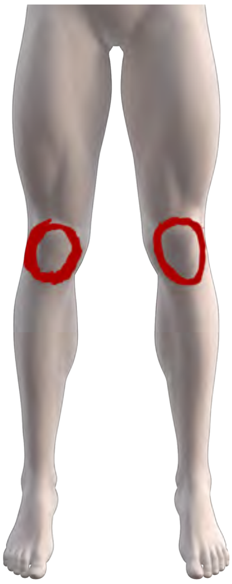

(g) Subject ID: 97

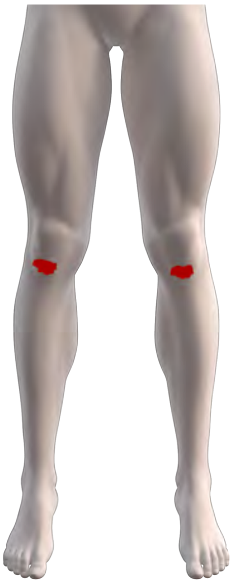

(h) Subject ID: 98

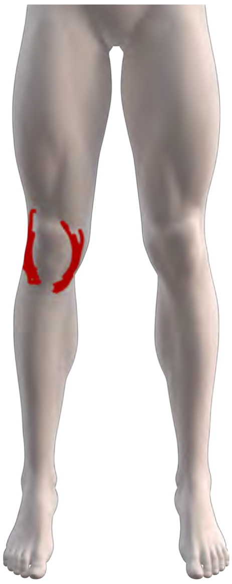

(i) Subject ID: 99

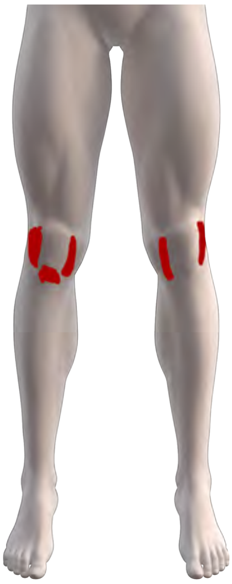

(a) Subject ID: 100

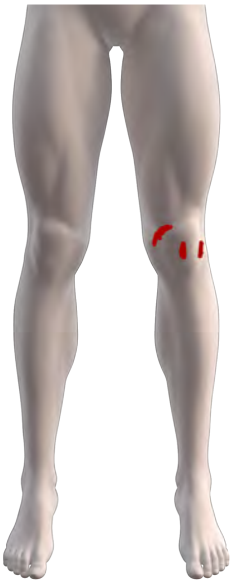

(b) Subject ID: 101

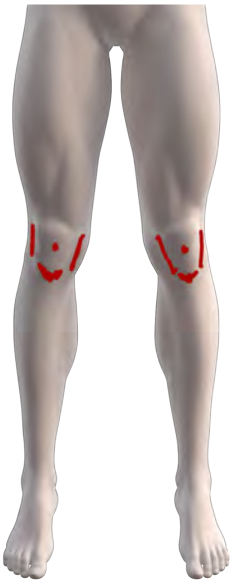

(c) Subject ID: 102

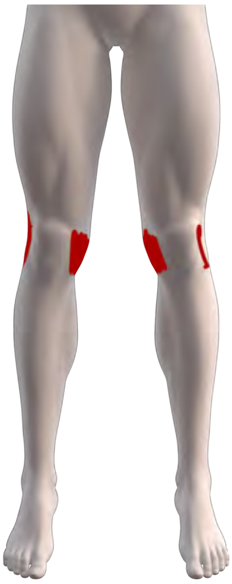

(d) Subject ID: 103

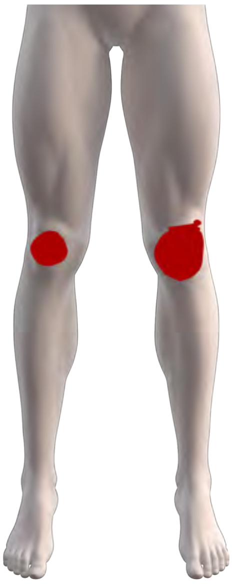

(e) Subject ID: 104

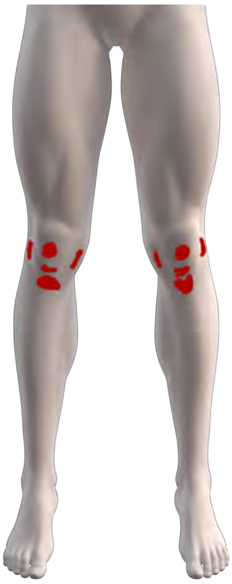

(f) Subject ID: 105

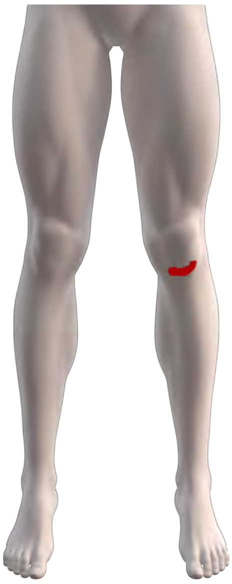

(g) Subject ID: 106

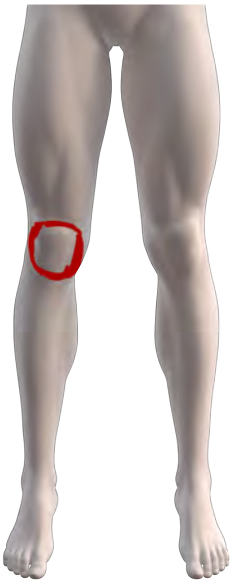

(h) Subject ID: 107

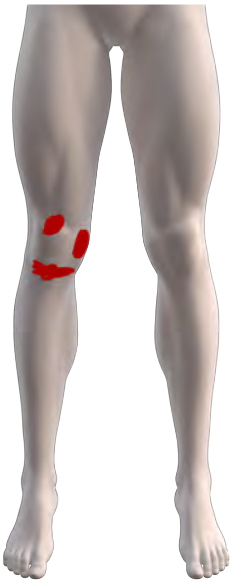

(i) Subject ID: 108

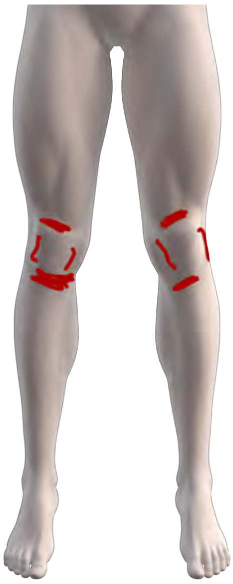

(a) Subject ID: 109

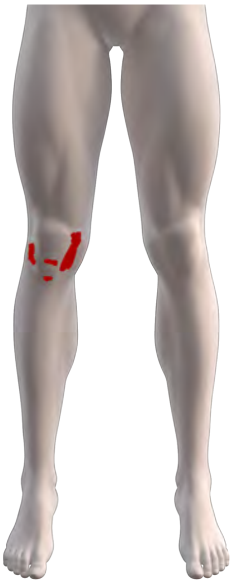

(b) Subject ID: 110

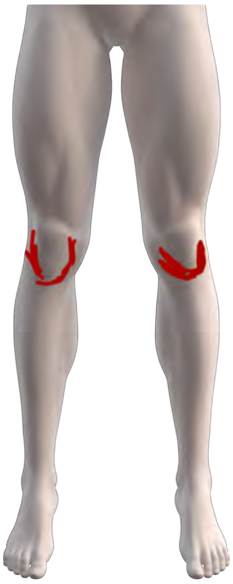

(c) Subject ID: 111

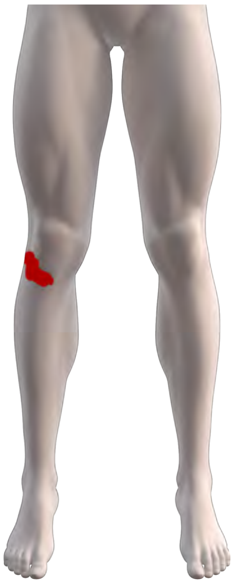

(d) Subject ID: 112

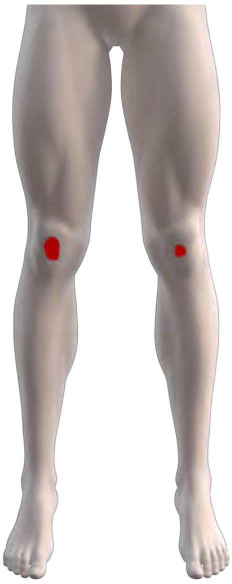

(e) Subject ID: 113

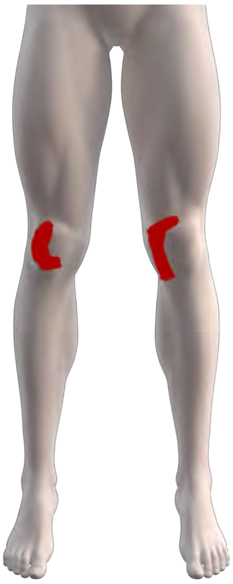

(f) Subject ID: 114

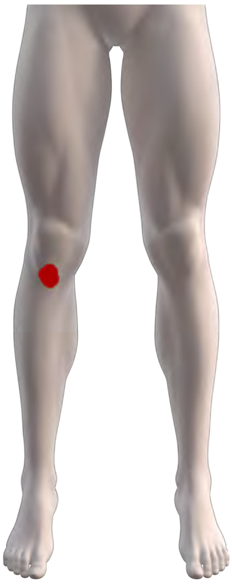

(g) Subject ID: 115

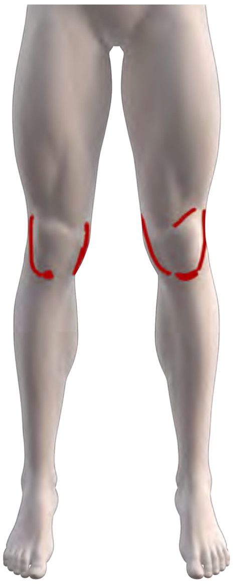

(h) Subject ID: 116

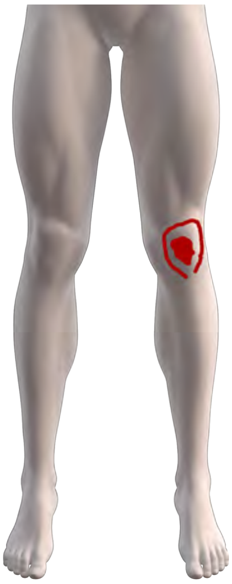

(i) Subject ID: 117

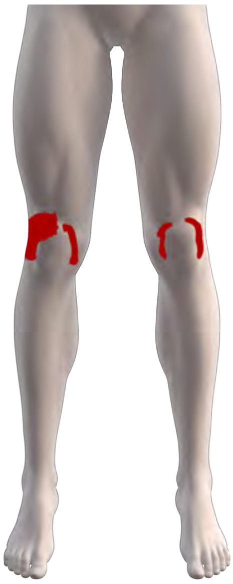

(a) Subject ID: 118

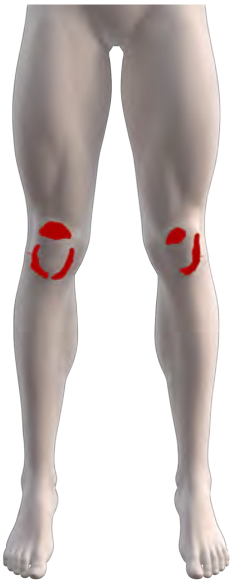

(b) Subject ID: 119

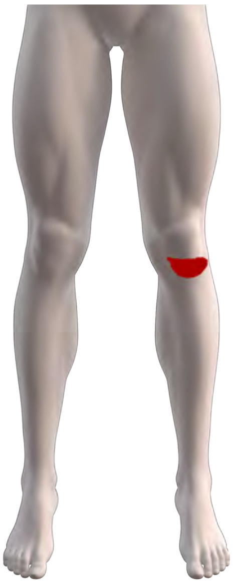

(c) Subject ID: 120

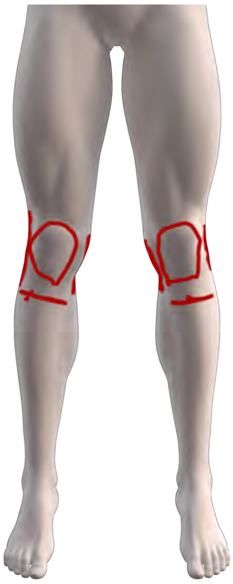

(d) Subject ID: 121

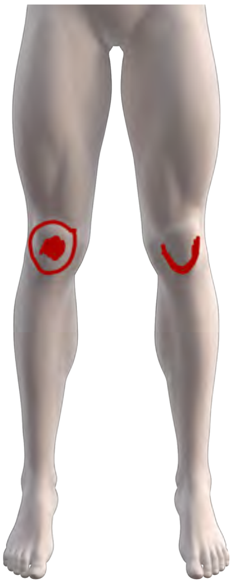

(e) Subject ID: 122

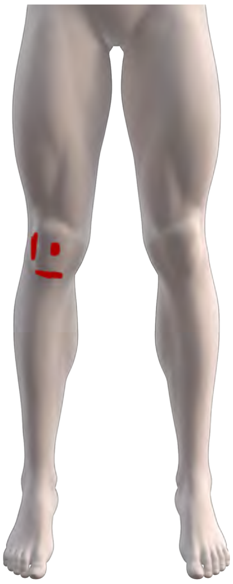

(f) Subject ID: 123

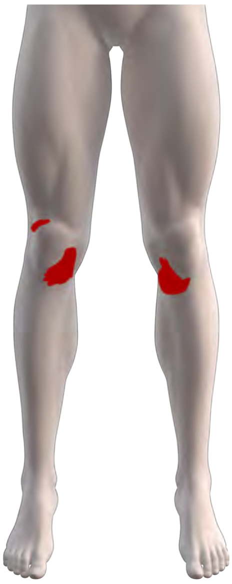

(g) Subject ID: 124

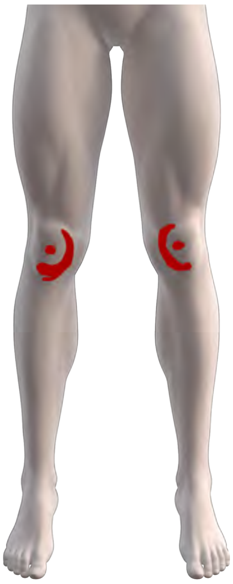

(h) Subject ID: 125

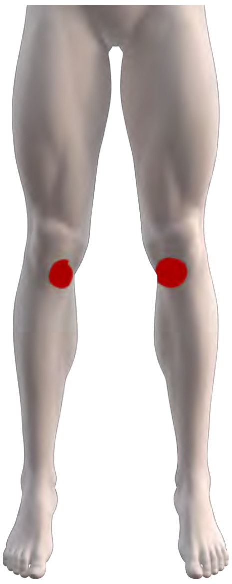

(i) Subject ID: 126

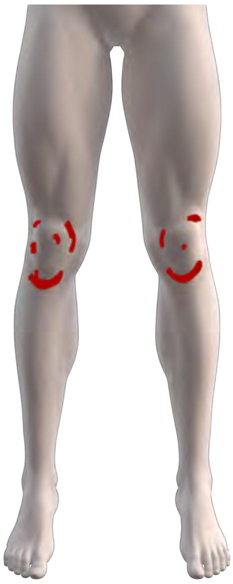

(a) Subject ID: 127

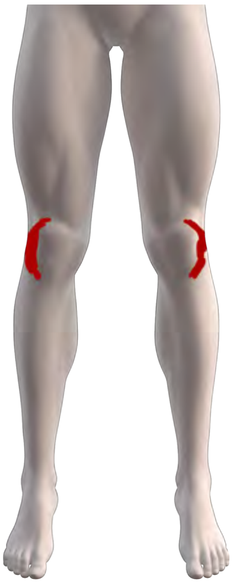

(b) Subject ID: 128

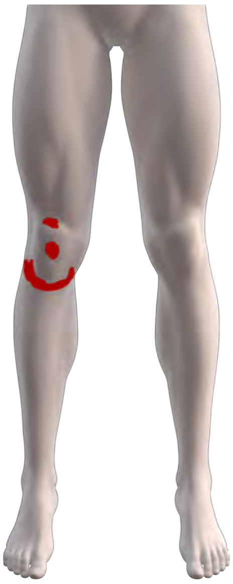

(c) Subject ID: 129

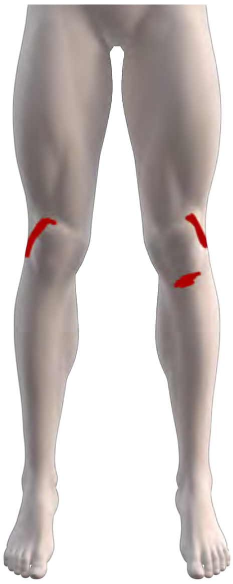

(d) Subject ID: 130

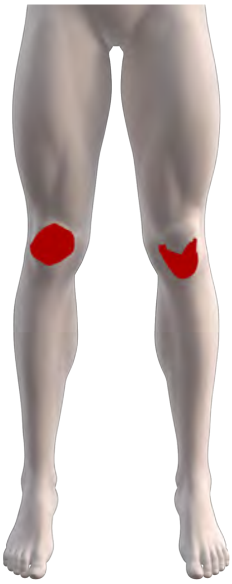

(e) Subject ID: 131

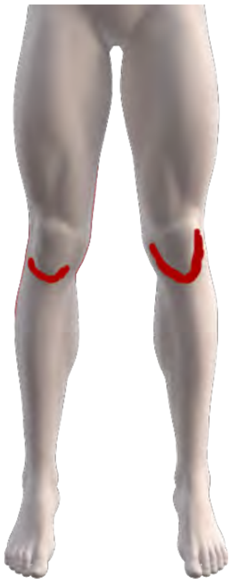

(f) Subject ID: 132

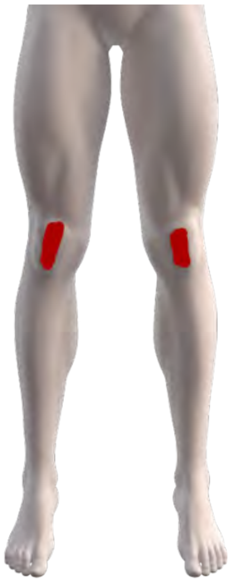

(g) Subject ID: 133

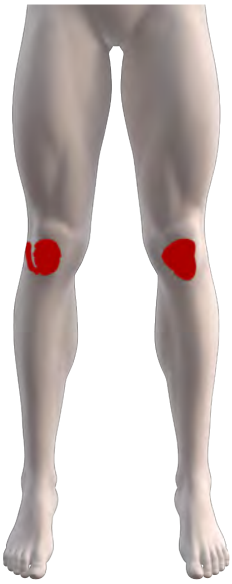

(h) Subject ID: 134

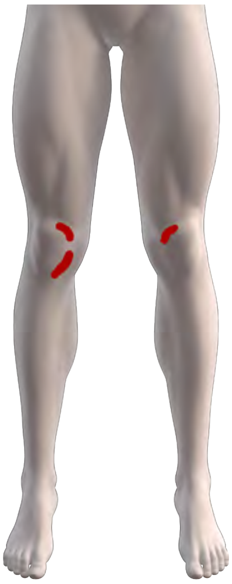

(i) Subject ID: 135

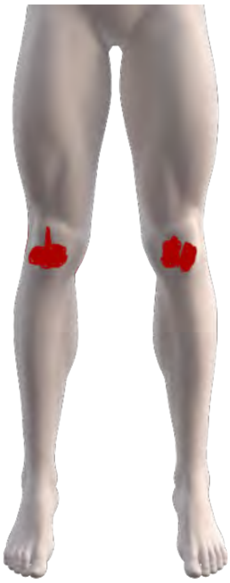

(a) *Subject ID: 136*

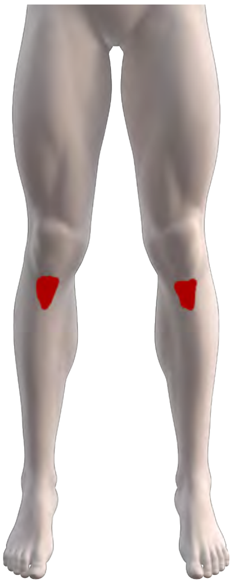

(b) *Subject ID: 137*

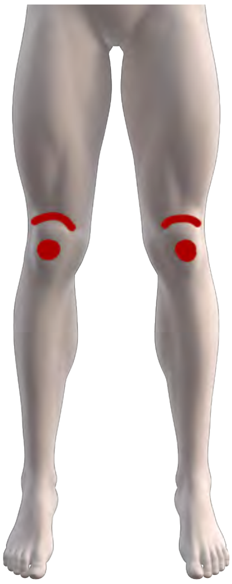

(c) *Subject ID: 138*

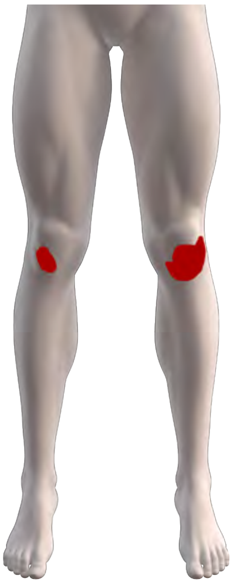

(d) *Subject ID: 139*

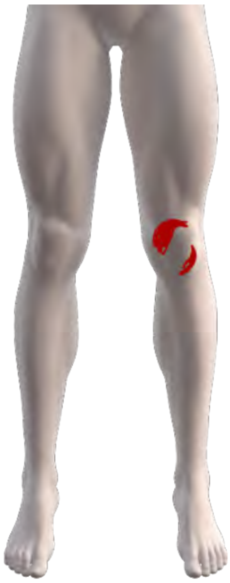

(e) *Subject ID: 140*

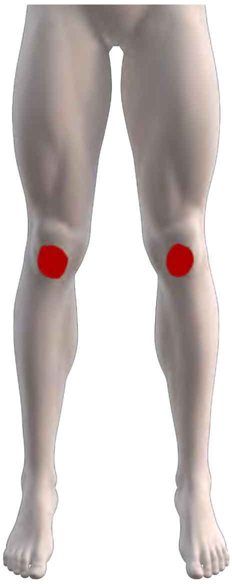

(f) *Subject ID: 141*

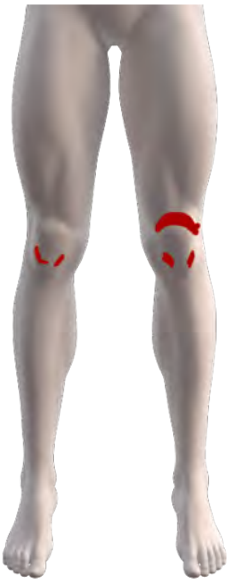

(g) *Subject ID: 142*

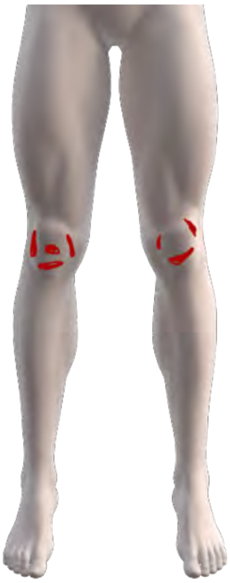

(h) *Subject ID: 143*

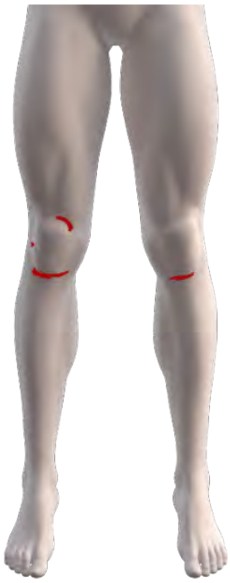

(i) *Subject ID: 144*

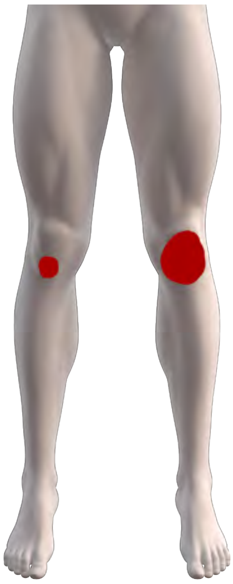

(a) Subject ID: 145

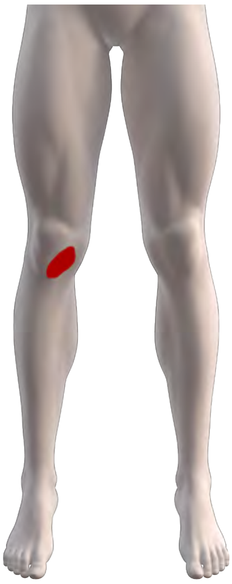

(b) Subject ID: 146

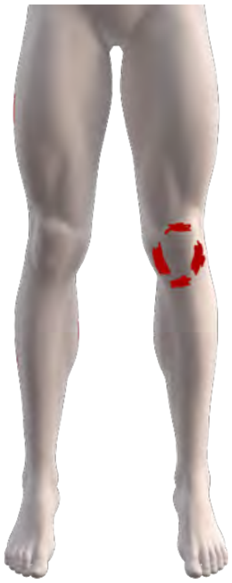

(c) Subject ID: 147

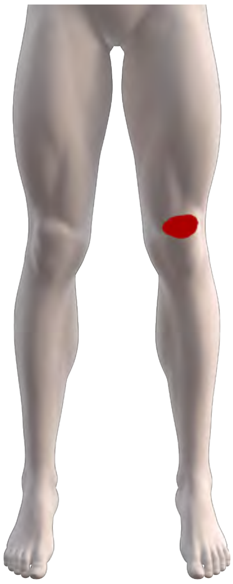

(d) Subject ID: 148

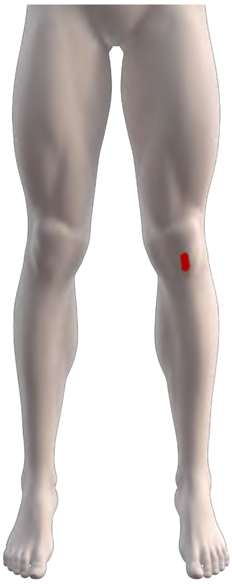

(e) Subject ID: 149

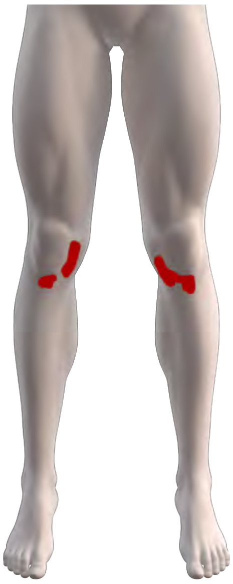

(f) Subject ID: 150

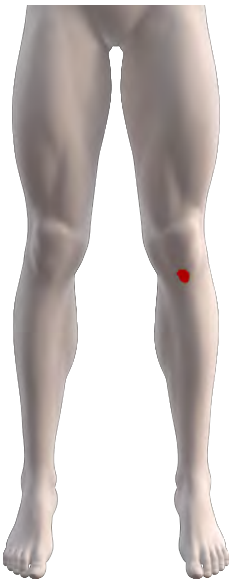

(g) Subject ID: 151

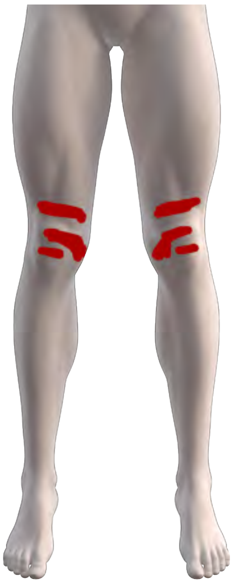

(h) Subject ID: 152

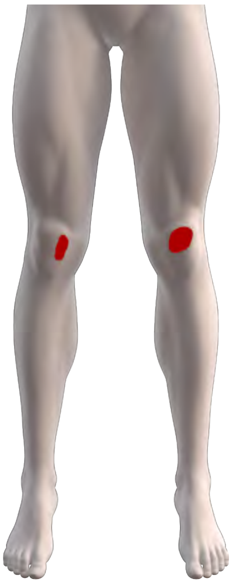

(i) Subject ID: 153

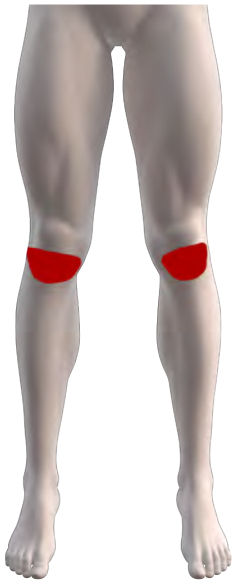

(a) Subject ID: 154

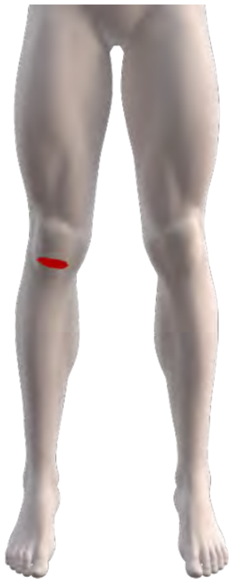

(b) Subject ID: 155

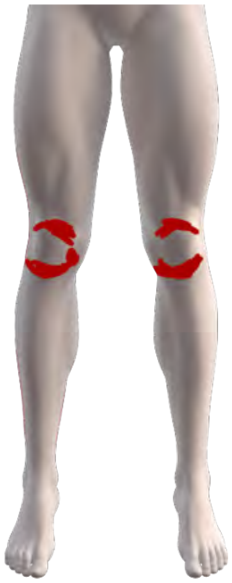

(c) Subject ID: 156

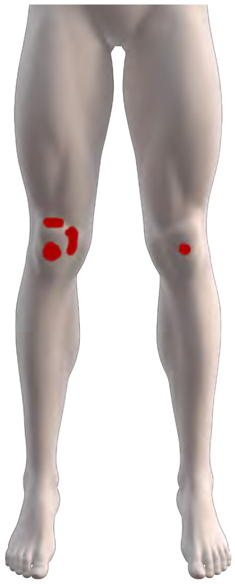

(d) Subject ID: 157

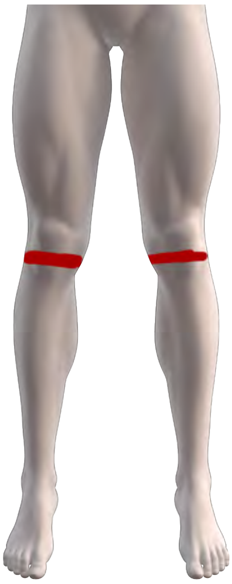

(e) Subject ID: 158

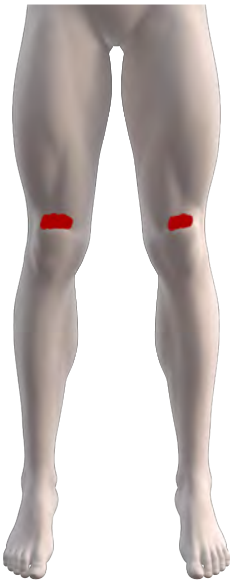

(f) Subject ID: 159

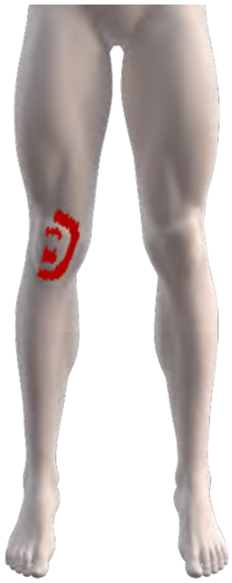

(g) Subject ID: 160

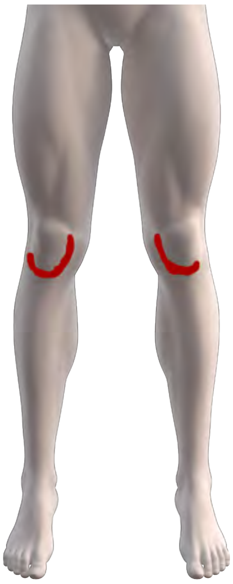

(h) Subject ID: 161

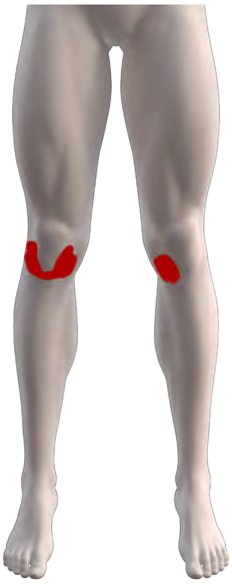

(i) Subject ID: 162

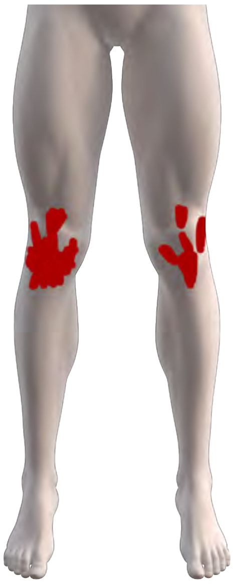

(a) Subject ID: 163

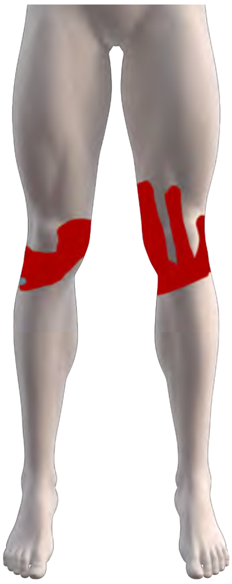

(b) Subject ID: 164

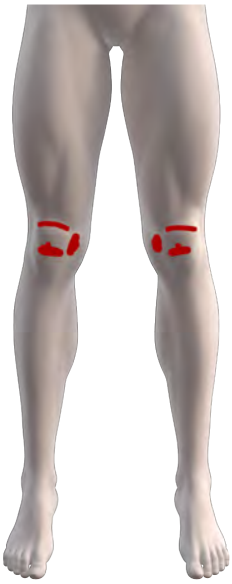

(c) Subject ID: 165

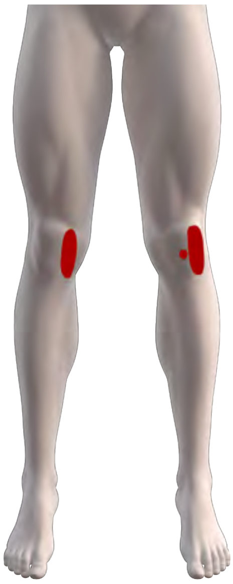

(d) Subject ID: 166

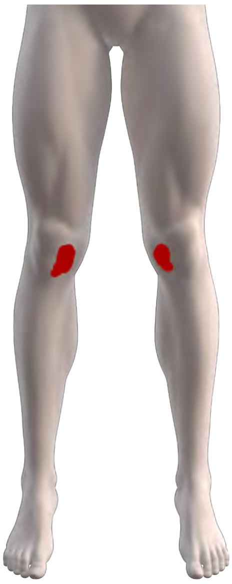

(e) Subject ID: 167

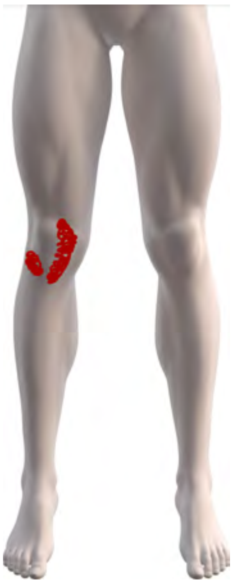

(f) Subject ID: 168

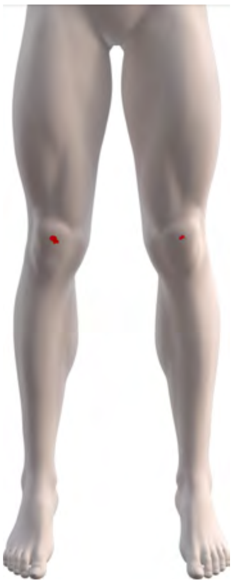

(g) Subject ID: 169

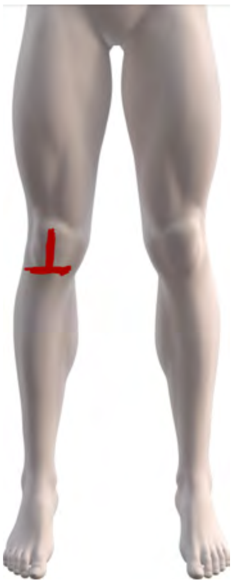

(h) Subject ID: 170

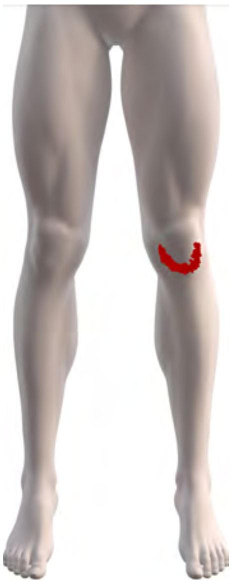

(i) Subject ID: 171

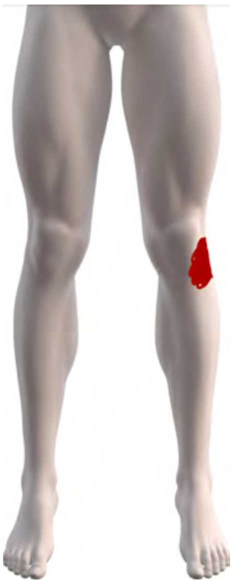

(a) Subject ID: 172

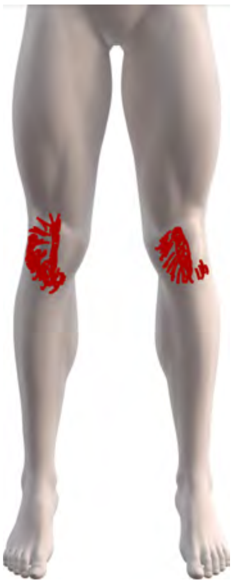

(b) Subject ID: 173

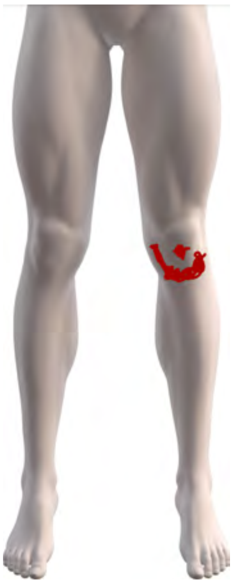

(c) Subject ID: 174

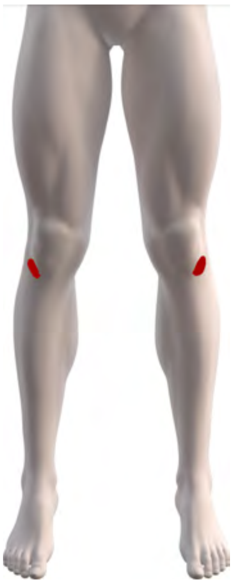

(d) Subject ID: 175

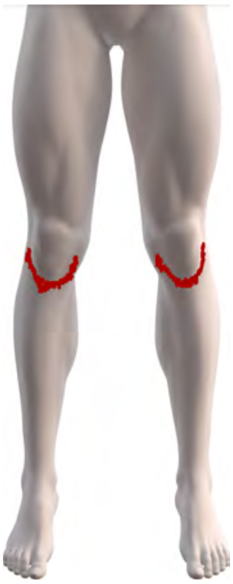

(e) Subject ID: 176

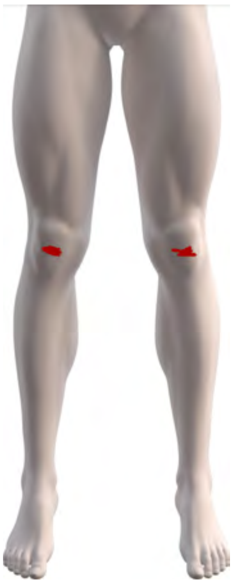

(f) Subject ID: 177

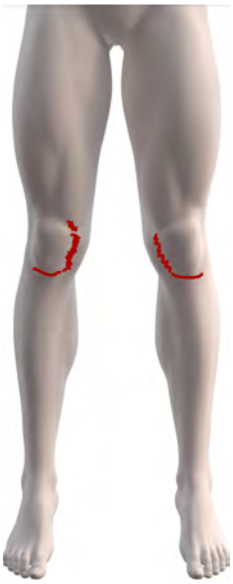

(g) Subject ID: 178

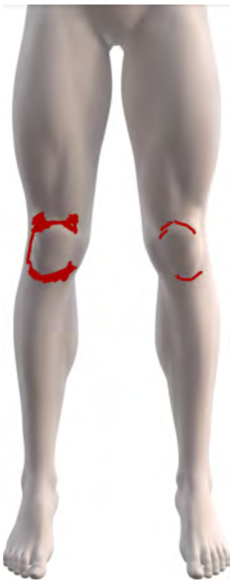

(h) Subject ID: 179

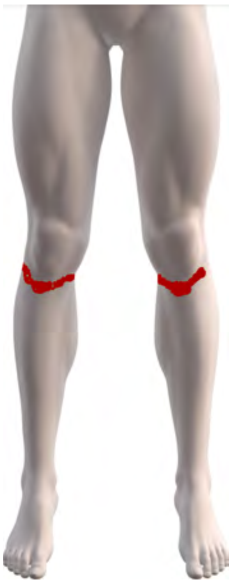

(i) Subject ID: 180

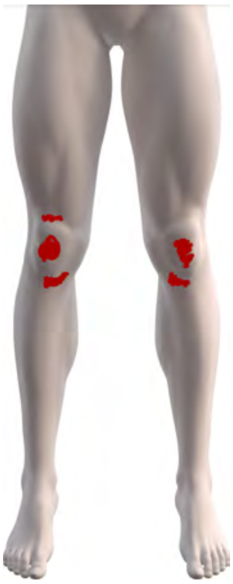

(a) Subject ID: 181

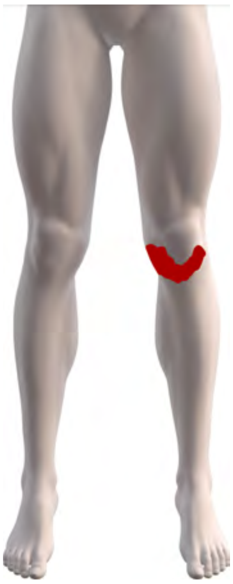

(b) Subject ID: 182

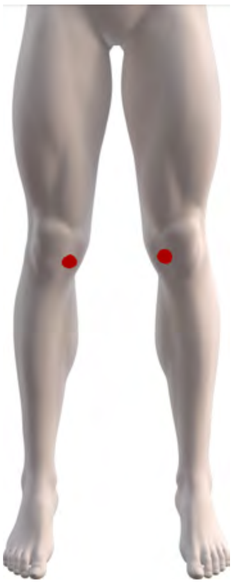

(c) Subject ID: 183

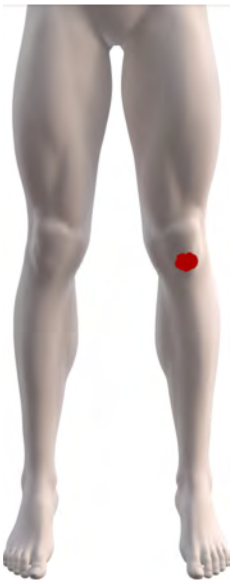

(d) Subject ID: 184

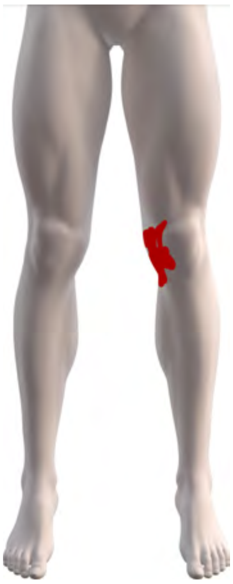

(e) Subject ID: 185

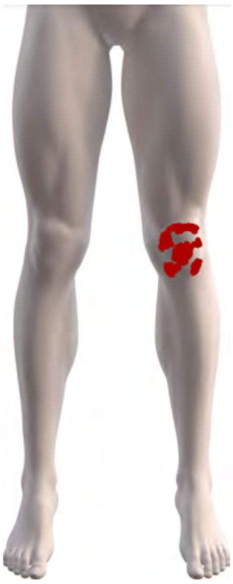

(f) Subject ID: 186

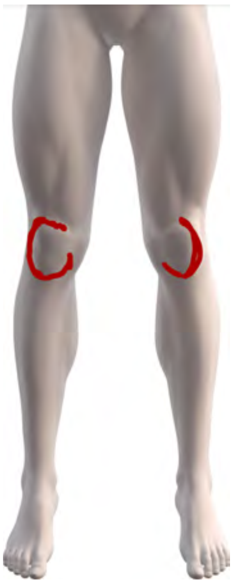

(g) Subject ID: 187

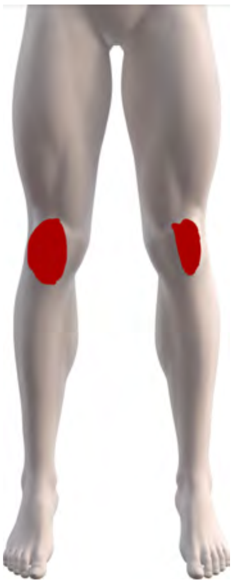

(h) Subject ID: 188

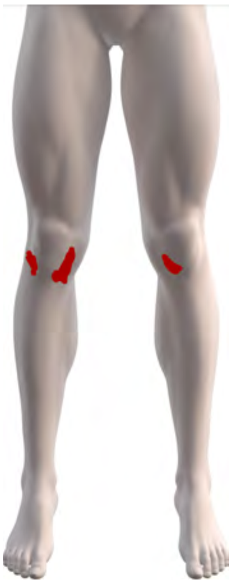

(i) Subject ID: 189

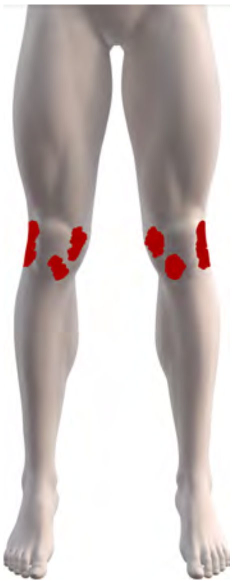

(a) Subject ID: 190

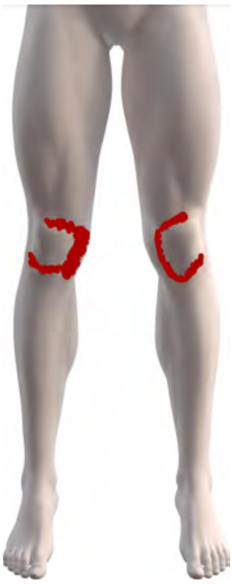

(b) Subject ID: 191

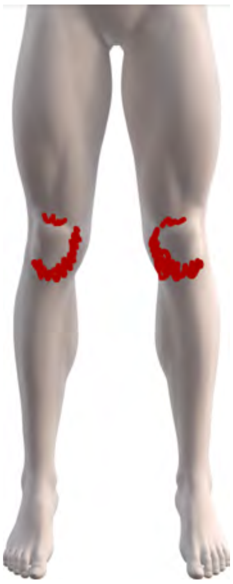

(c) Subject ID: 192

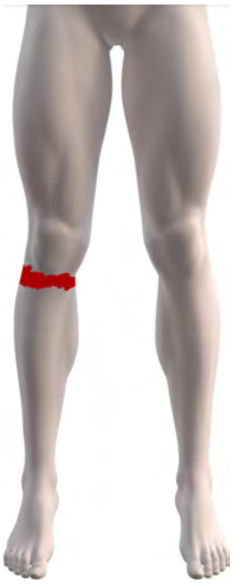

(d) Subject ID: 193

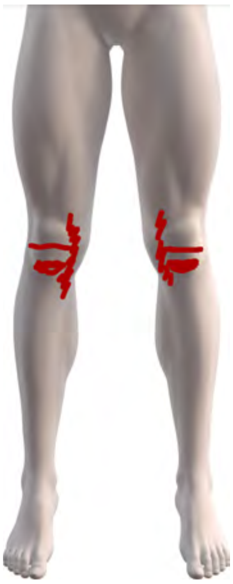

(e) Subject ID: 194

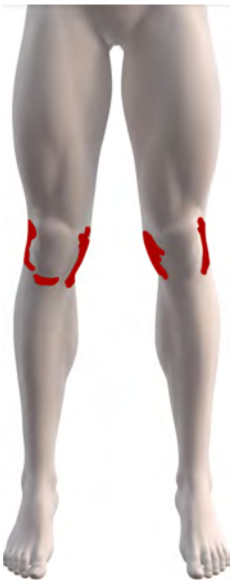

(f) Subject ID: 195

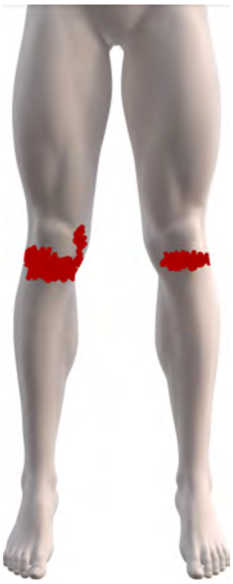

(g) Subject ID: 196

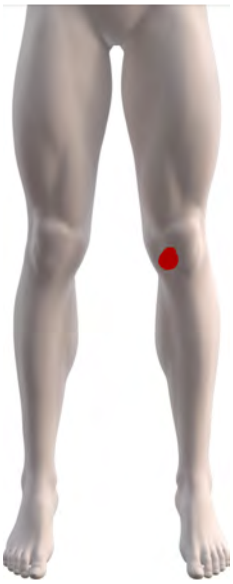

(h) Subject ID: 197

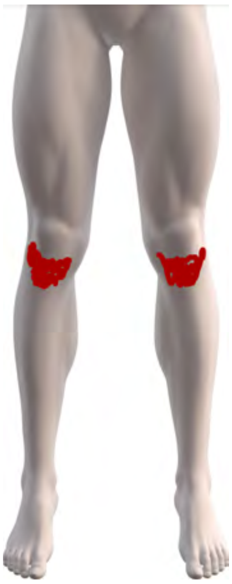

(i) Subject ID: 198

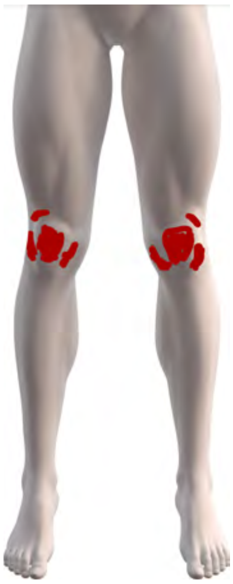

(a) Subject ID: 199

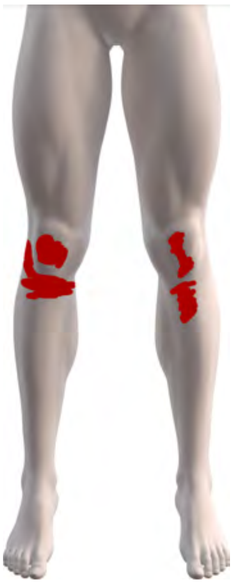

(b) Subject ID: 200

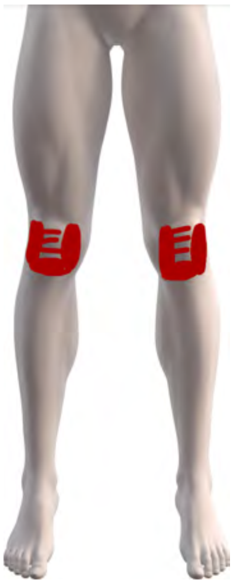

(c) Subject ID: 201

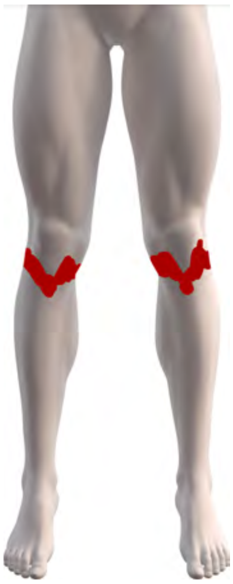

(d) Subject ID: 202

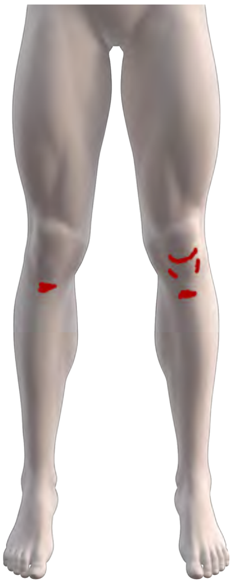

(e) Subject ID: 203

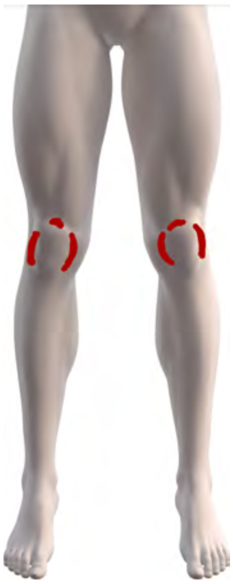

(f) Subject ID: 204

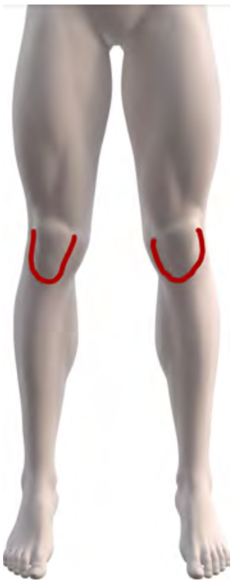

(g) Subject ID: 205

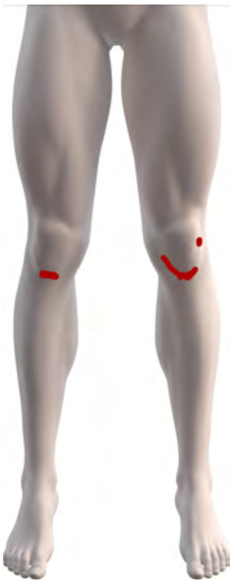

(h) Subject ID: 206

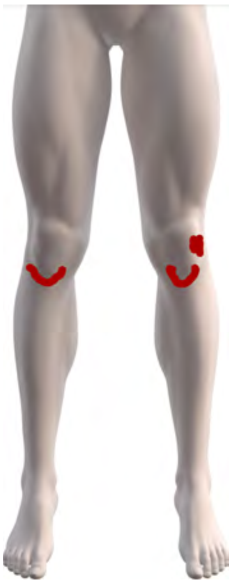

(i) Subject ID: 207

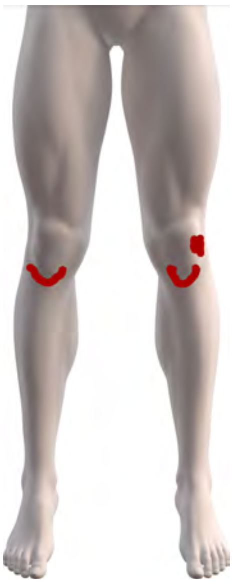
(a) Subject ID: 208

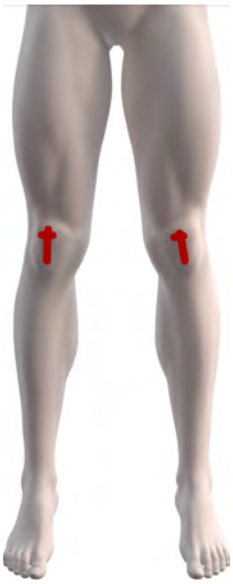
(b) Subject ID: 209

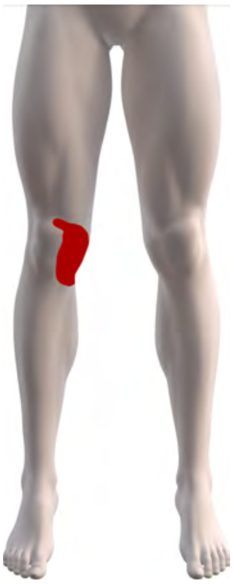
(c) Subject ID: 210

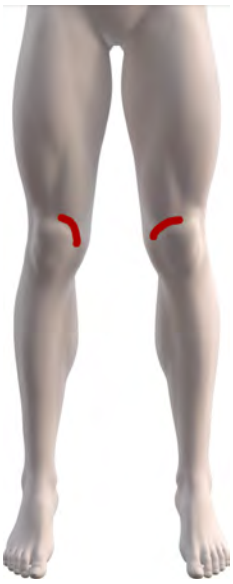
(d) Subject ID: 211

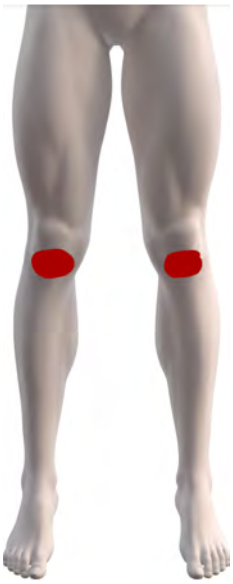
(e) Subject ID: 212

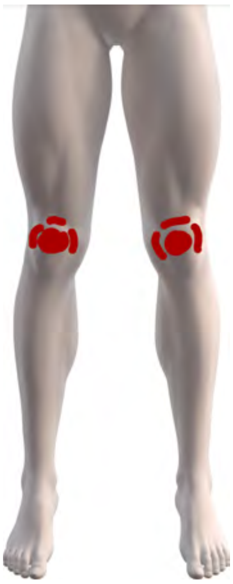
(f) Subject ID: 213

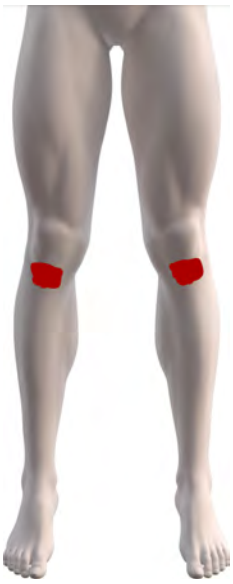
(g) Subject ID: 214

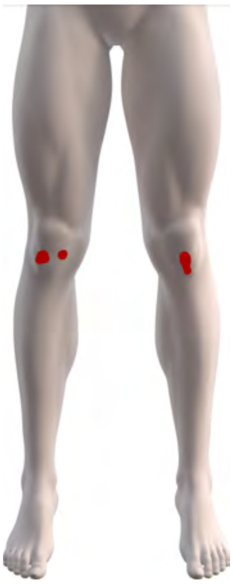
(h) Subject ID: 215

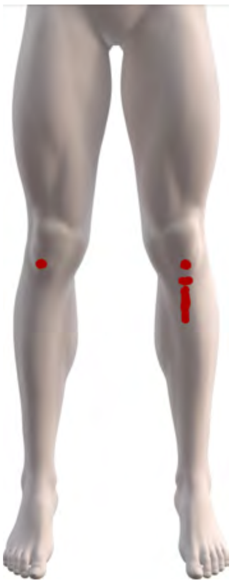
(i) Subject ID: 216

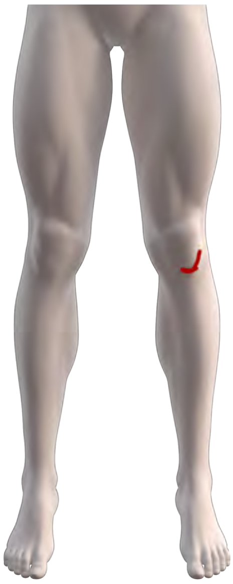
(a) Subject ID: 217

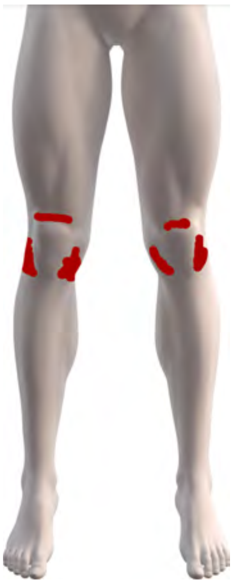
(b) Subject ID: 218

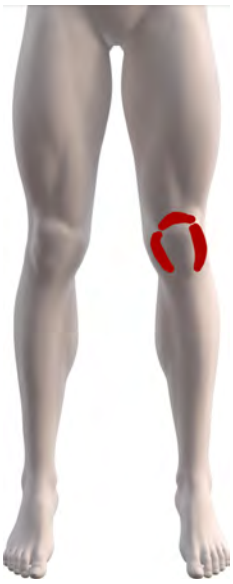
(c) Subject ID: 219

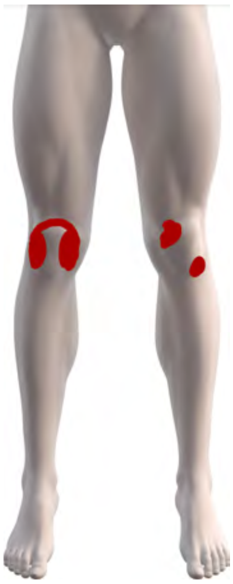
(d) Subject ID: 220

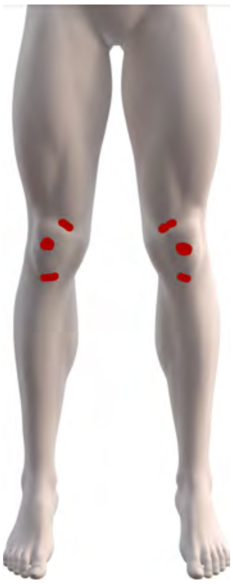
(e) Subject ID: 221

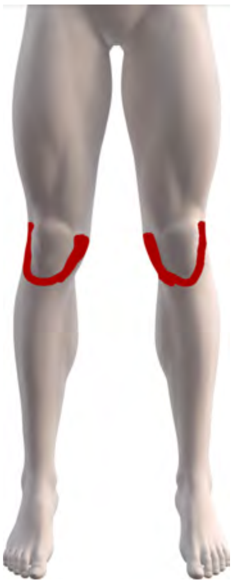
(f) Subject ID: 222

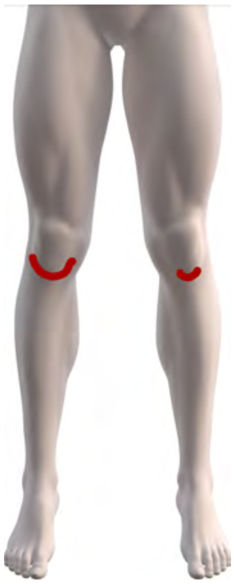
(g) Subject ID: 223

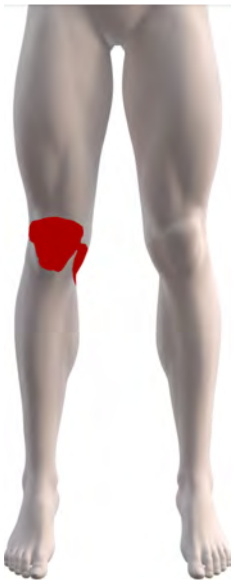
(h) Subject ID: 224

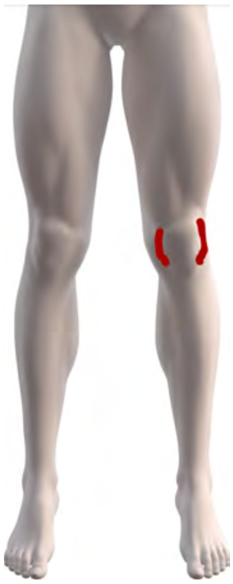
(i) Subject ID: 225

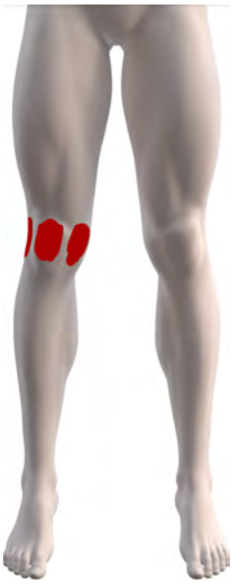

(a) Subject ID: 226

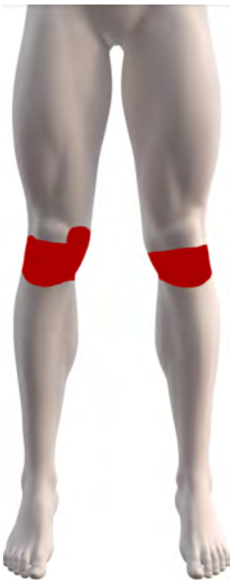

(b) Subject ID: 227

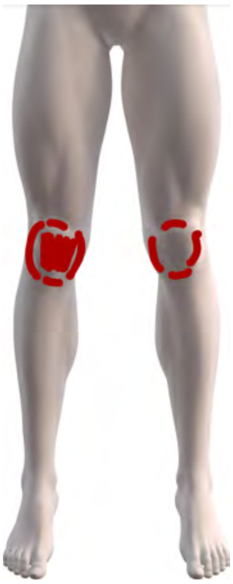

(c) Subject ID: 228

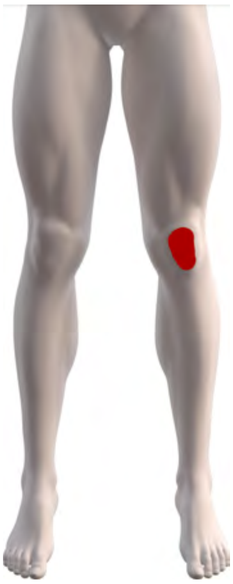

(d) Subject ID: 229

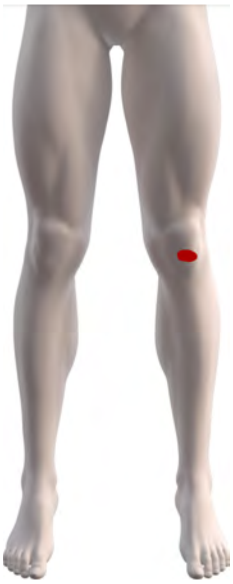

(e) Subject ID: 230

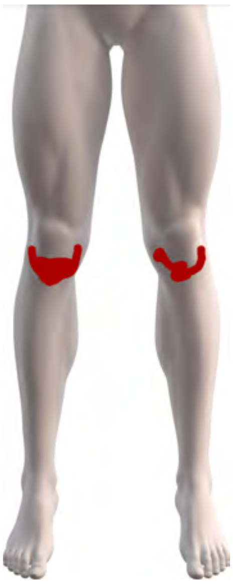

(f) Subject ID: 231

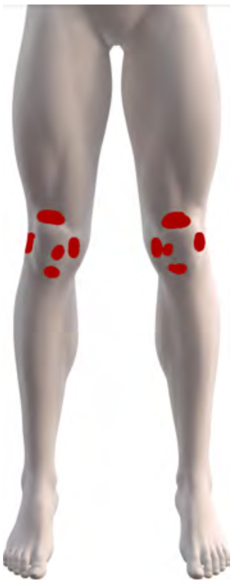

(g) Subject ID: 232

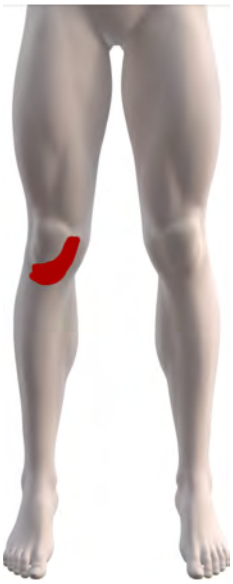

(h) Subject ID: 233

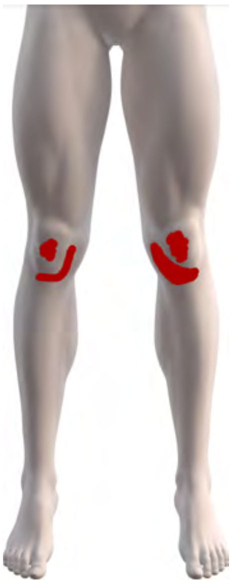

(i) Subject ID: 234

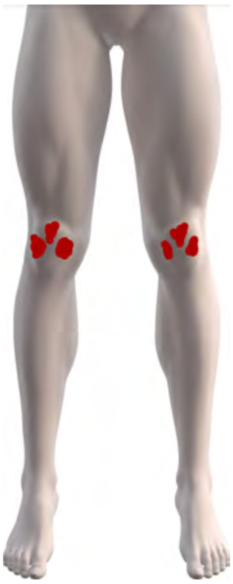
(a) Subject ID: 235

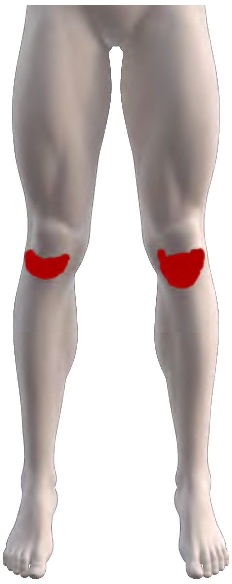
(b) Subject ID: 236

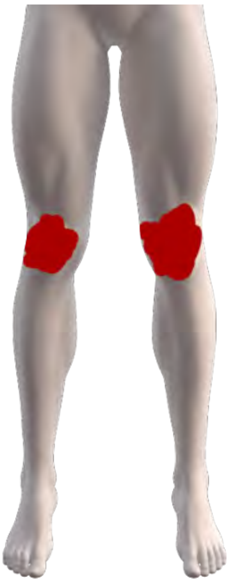
(c) Subject ID: 237

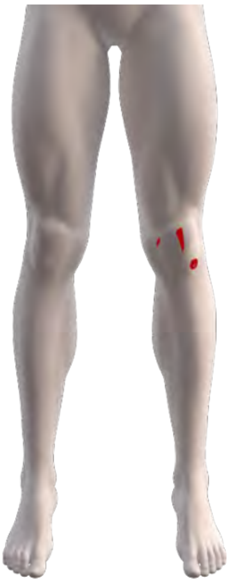
(d) Subject ID: 238

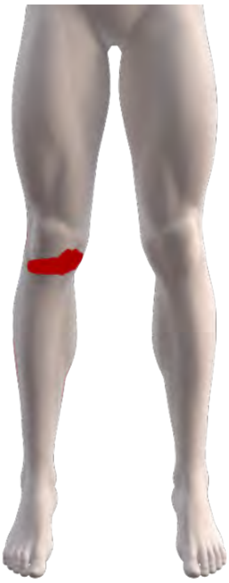
(e) Subject ID: 239

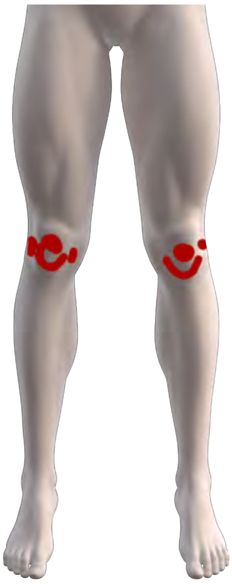
(f) Subject ID: 240

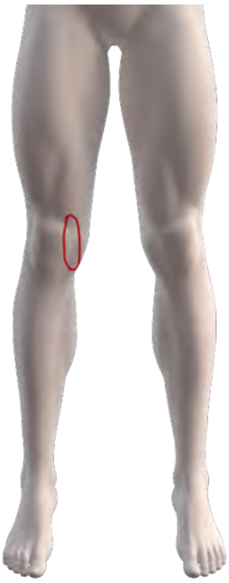
(g) Subject ID: 241

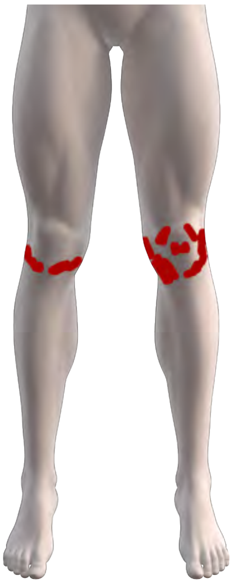
(h) Subject ID: 242

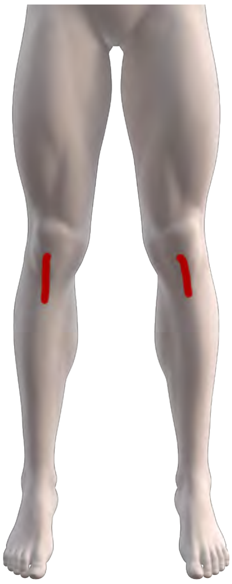
(i) Subject ID: 243

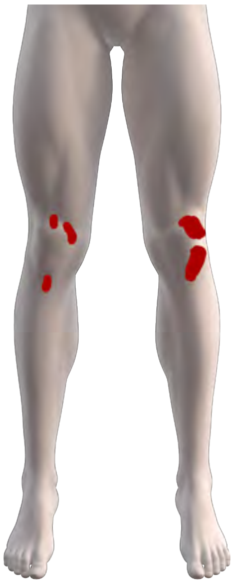
(a) Subject ID: 244

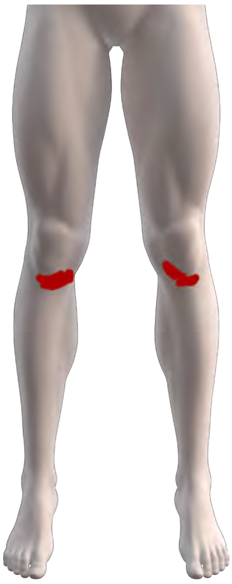
(b) Subject ID: 245

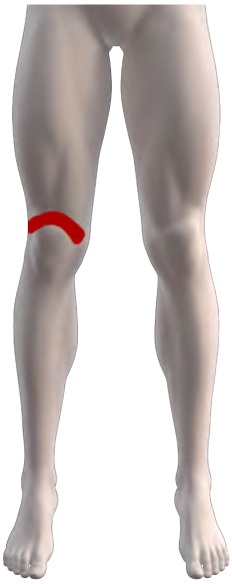
(c) Subject ID: 246

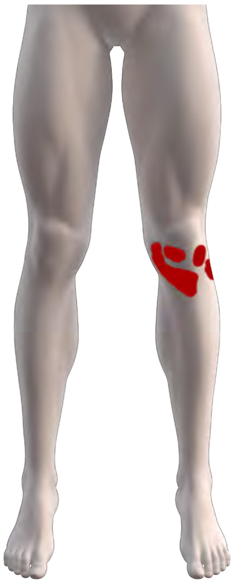
(d) Subject ID: 247

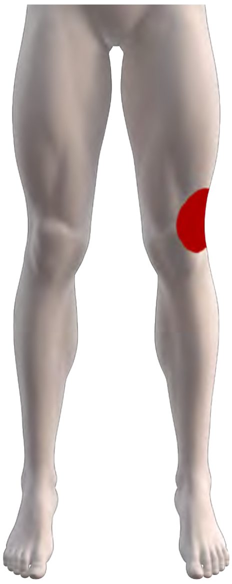
(e) Subject ID: 248

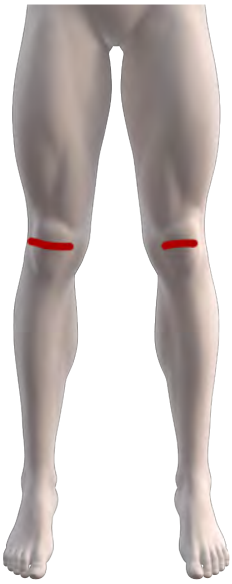
(f) Subject ID: 249

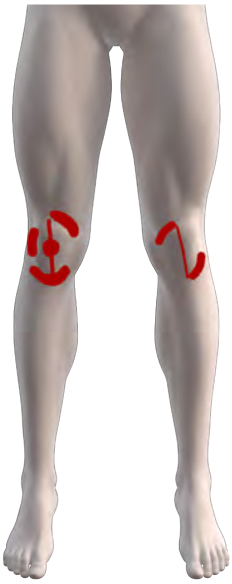
(g) Subject ID: 250

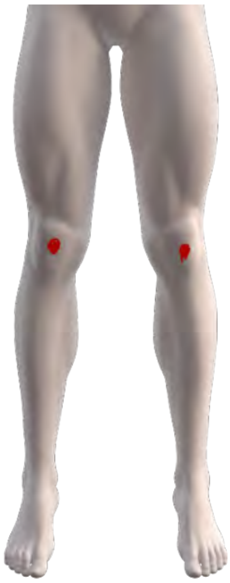
(h) Subject ID: 251

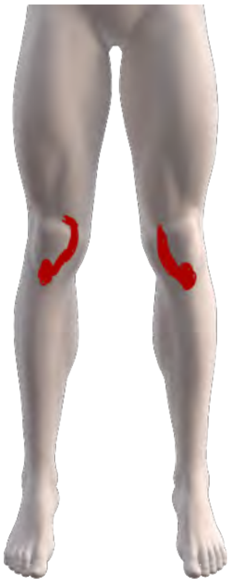
(i) Subject ID: 252

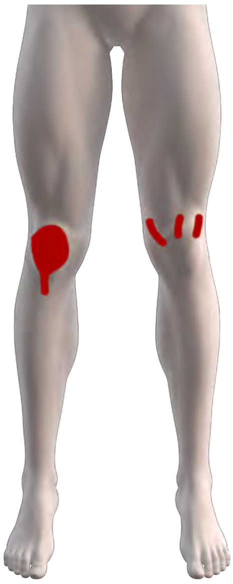

(a) Subject ID: 253

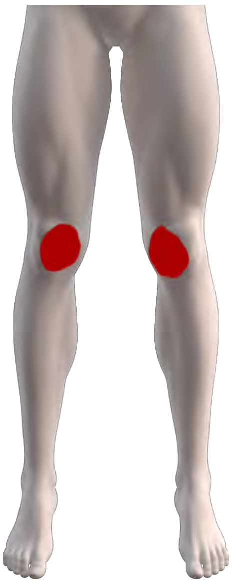

(b) Subject ID: 254

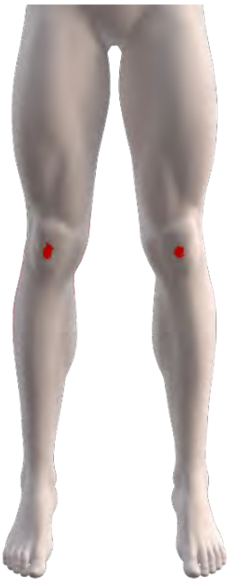

(c) Subject ID: 255

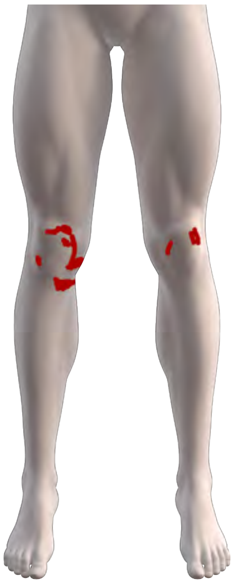

(d) Subject ID: 256

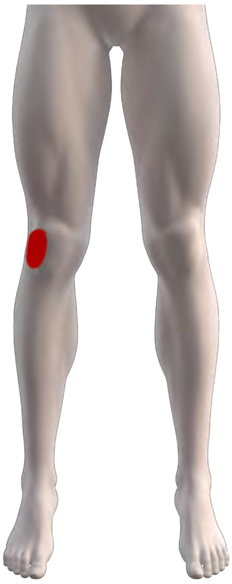

(e) Subject ID: 257

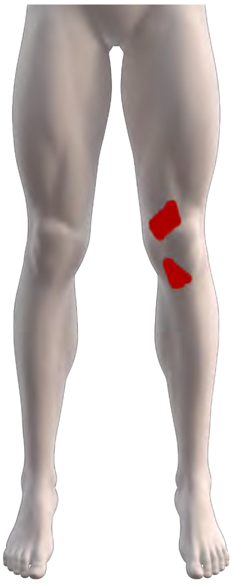

(f) Subject ID: 258

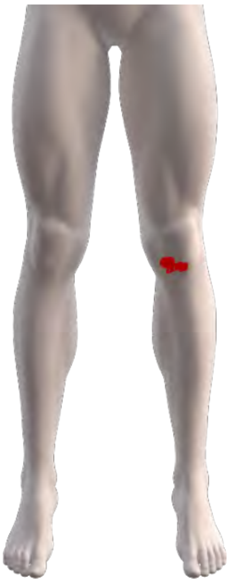

(g) Subject ID: 259

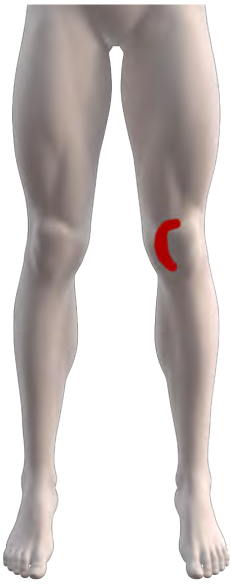

(h) Subject ID: 260

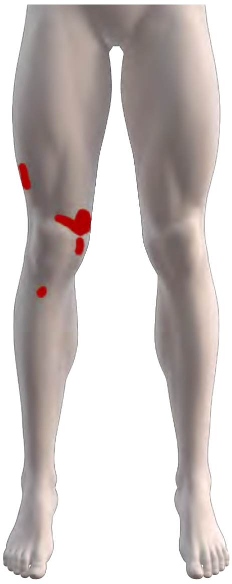

(i) Subject ID: 261

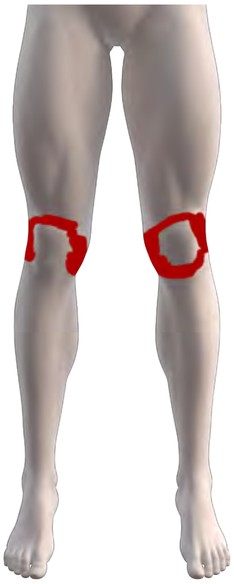

(a) Subject ID: 262

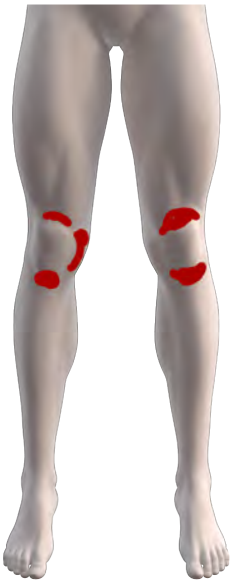

(b) Subject ID: 263

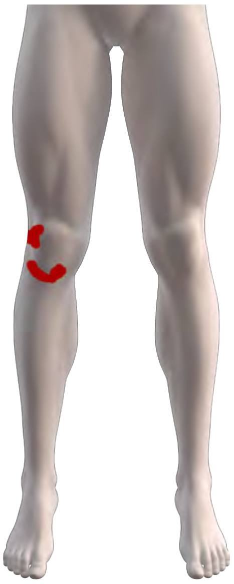

(c) Subject ID: 264

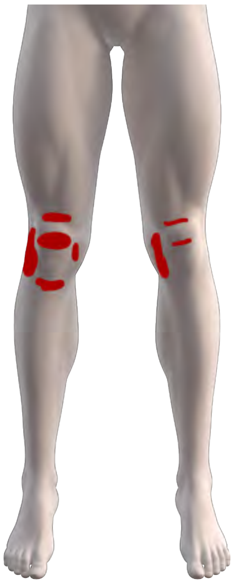

(d) Subject ID: 265

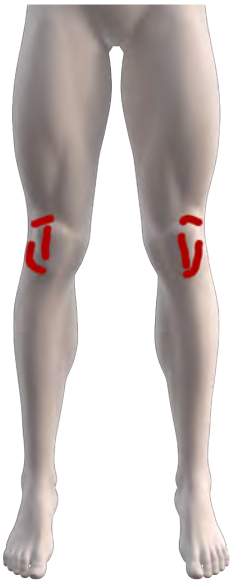

(e) Subject ID: 266

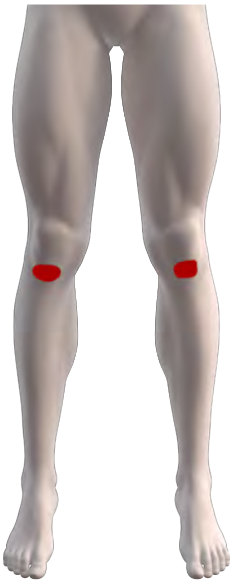

(f) Subject ID: 267

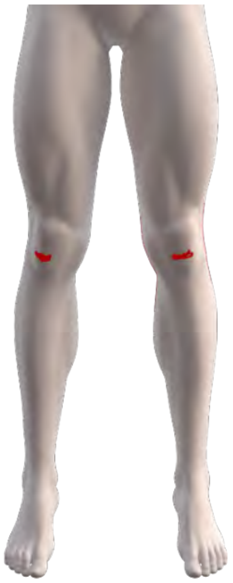

(g) Subject ID: 268

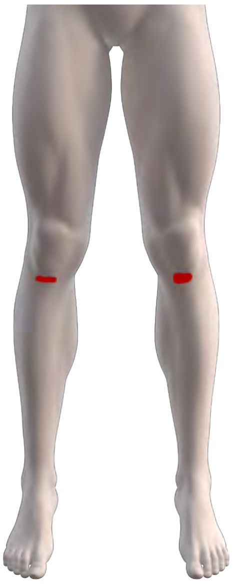

(h) Subject ID: 269

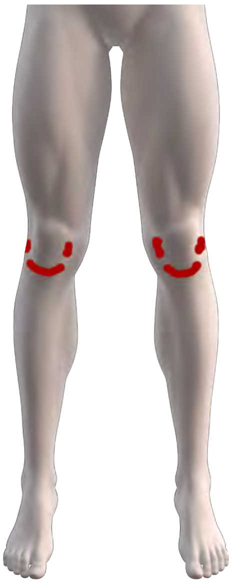

(i) Subject ID: 270

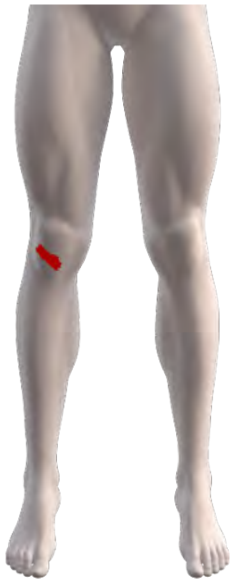
(a) Subject ID: 271

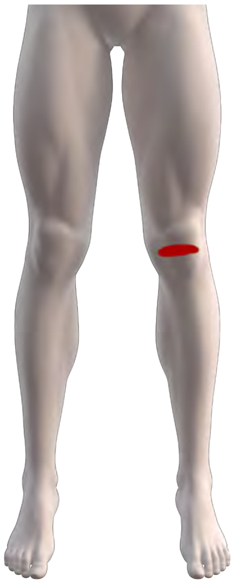
(b) Subject ID: 272

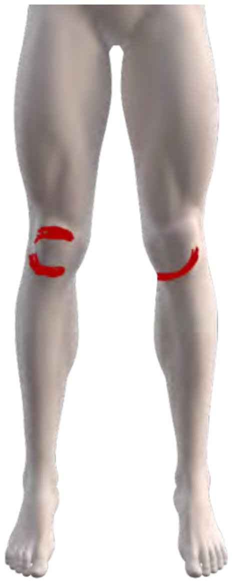
(c) Subject ID: 273

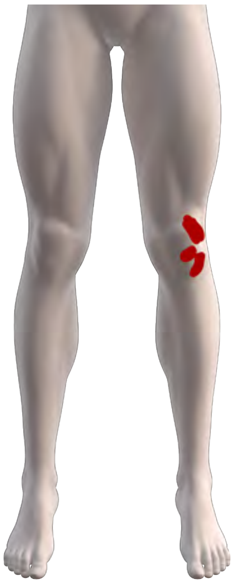
(d) Subject ID: 274

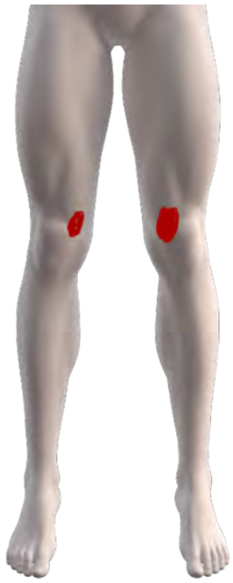
(e) Subject ID: 275

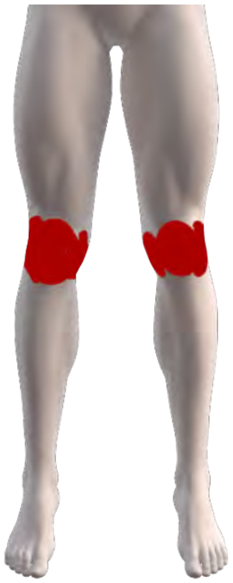
(f) Subject ID: 276

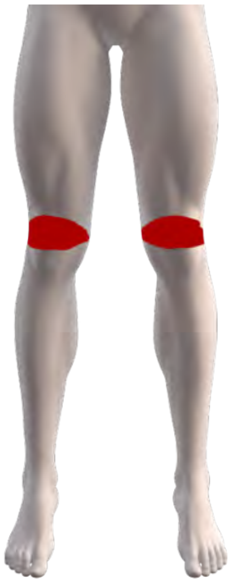
(g) Subject ID: 277

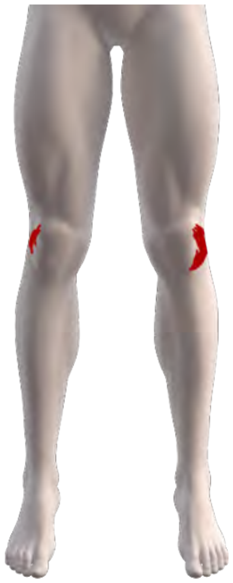
(h) Subject ID: 278

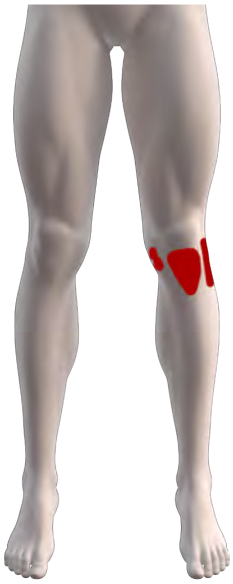
(i) Subject ID: 279

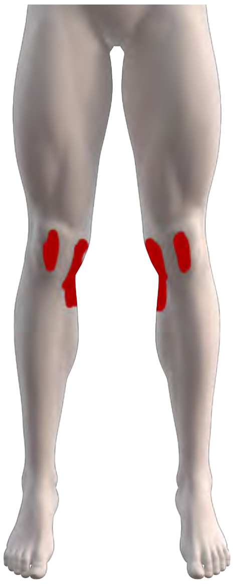

(a) Subject ID: 280

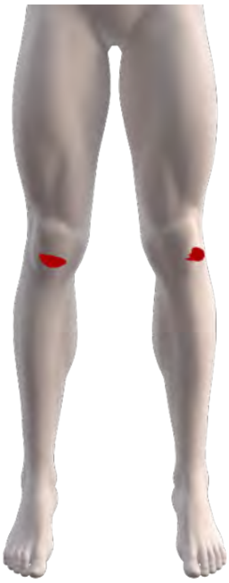

(b) Subject ID: 281

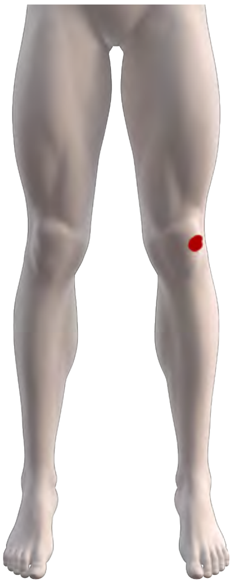

(c) Subject ID: 282

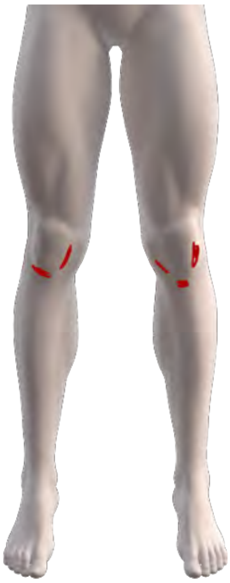

(d) Subject ID: 283

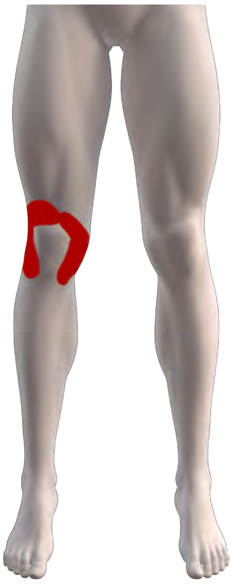

(e) Subject ID: 284

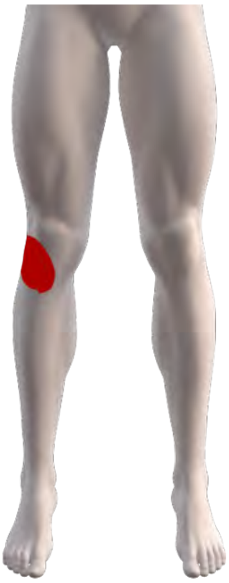

(f) Subject ID: 285

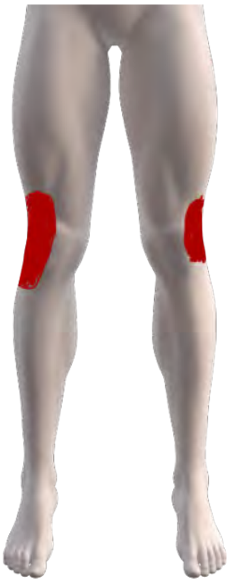

(g) Subject ID: 286

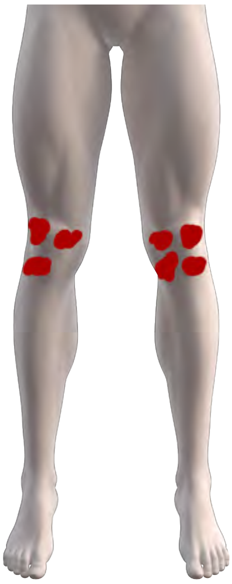

(h) Subject ID: 287

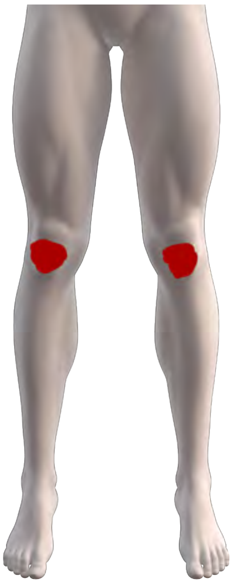

(i) Subject ID: 288

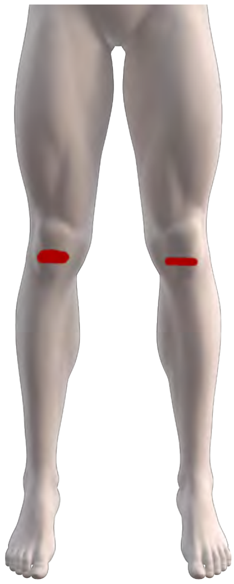

(a) Subject ID: 289

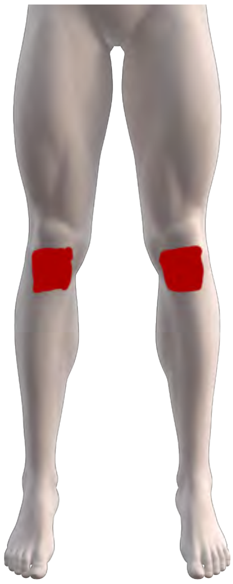

(b) Subject ID: 290

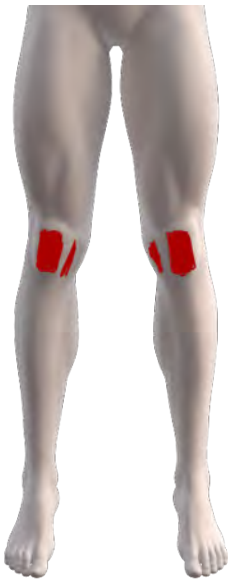

(c) Subject ID: 291

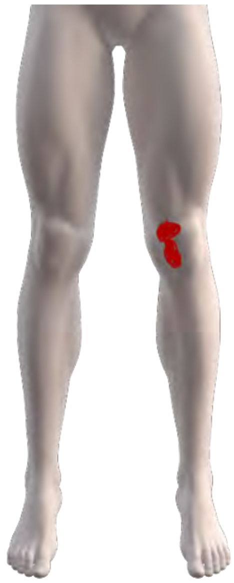

(d) Subject ID: 292

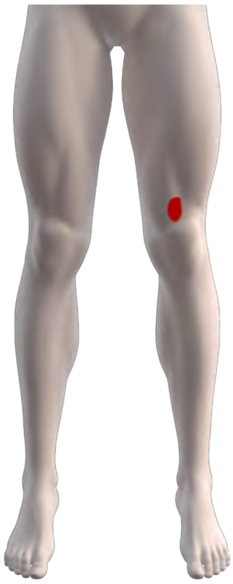

(e) Subject ID: 293

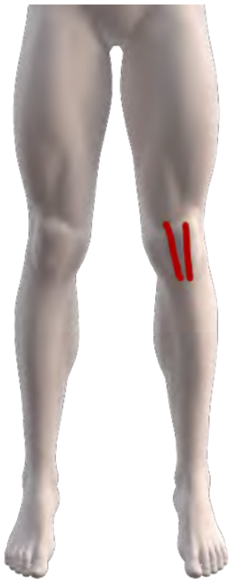

(f) Subject ID: 294

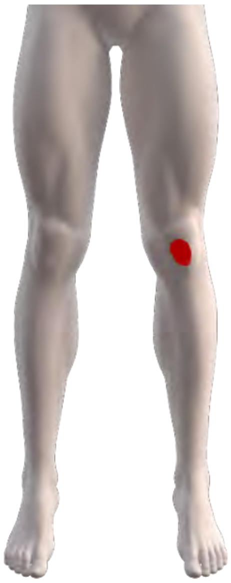

(g) Subject ID: 295

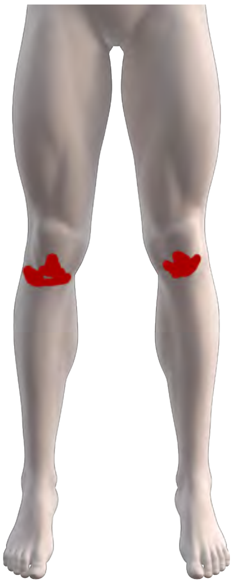

(h) Subject ID: 296

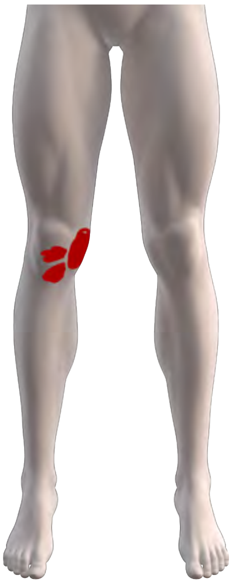

(i) Subject ID: 297

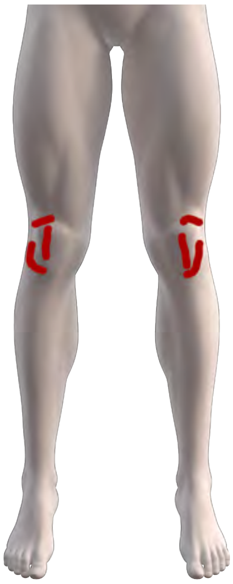

(a) Subject ID: 298

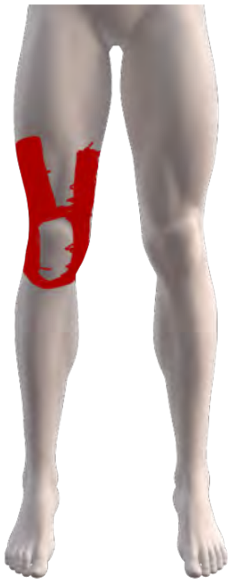

(b) Subject ID: 299
